# IGF2BP2-m6A-circMMP9 axis recruits ETS1 to promote TRIM59 transcription in laryngeal squamous cell carcinoma

**DOI:** 10.1038/s41598-024-53422-4

**Published:** 2024-02-06

**Authors:** Jinling Li, Huan Cao, Jianwang Yang, Baoshan Wang

**Affiliations:** Department of Otorhinolaryngology, The Second Hospital of Hebei Medical University, Shijiazhuang, Hebei China

**Keywords:** Cancer, Cell biology

## Abstract

Laryngeal squamous cell carcinoma (LSCC) is a common malignancy of the head and neck. Recently, circular RNA (circRNA) has been studied extensively in multisystem diseases. However, there are few research on biological functions and molecular mechanisms of circRNAs in LSCC. CircRNA array was used to detect the differentially expressed circRNAs. Kaplan–Meier and cox regression analysis were used to identify survival based on circMMP9. The qRT-PCR, RNase R treatment, sanger sequencing and in situ hybridization were used to verify circMMP9 expression, characteristics and localization in LSCC tissues and cells. Functionally, colony formation, MTS, transwell and in vivo assays were proceeded to detect the biological function of circMMP9 in LSCC progression. The RNA-seq was conducted to identify the molecular targets of circMMP9. Mechanically, MeRIP, RNA Immunoprecipitation (RIP), RNA pulldown, Chromatin immunoprecipitation (ChIP) and dual-luciferase reporter assays were carried on to verify the regulatory mechanism of circMMP9. CircMMP9 was discovered upregulated in LSCC tissues and cells, and high level of circMMP9 was associated with poor prognosis, low degree of pathological grading, high TNM stage and lymph node metastasis of LSCC. CircMMP9 knockdown prevented LSCC progression both in vitro and in vivo, whereas, circMMP9 overexpression had the opposite effect. CircMMP9 was stabilized by IGF2BP2 in m6A-dependent manner. TRIM59 was identified as downstream target of circMMP9. CircMMP9 recruited ETS1 to stimulate TRIM59 transcription. Moreover, TRIM59 accelerated LSCC progression via activating the PI3K/AKT signal pathway. Our findings offered a unique regulatory mechanism for circMMP9 in LSCC, as well as a novel proof that circMMP9 may be utilize as a diagnostic marker and therapeutic target for LSCC patients.

## Introduction

Laryngeal squamous cell carcinoma (LSCC), as a common malignancy of the head and neck, causes in approximately 160,420 deaths each year in the United States^1,2^. Also, the functions of vocal, respiratory and swallowing with LSCC patients are severely impaired^3^. Due to the vague mechanisms of the progression, the prognosis of metastatic status in LSCC is poor^4,5^. Thus, it is vitally important to study the molecular mechanisms of advanced LSCC for exploring novel detection markers or advanced therapeutic strategies.

As novel RNAs, circRNAs are of expression stability and tissue specificity^6^, and plenty of circRNAs are discovered dysregulated in cancers by emerging studies^7,8^. The characteristics of circRNAs determine they may act as potential diagnostic markers and targets for malignancies. As potentially novel biomarkers, circRNAs were responsible for tumor progression^9^. In NSCLC, circRNAs served as diagnostic and prognostic biomarkers^10^. However, in LSCC, detecting potential diagnostic markers and therapeutic targets is a matter of some urgency. Mechanically, circRNAs functioned as sponge for miRNAs, scaffolds for proteins, templates for translation, and regulators of mRNA transcription and stability^11^. Also, there was a new role for circRNAs in regulating gene expression in the nucleus^12^. In gastric cancer, circMRPS35 recruited KAT7 to promote FOXO1/3a transcription suppressing malignant progression^13^. In breast cancer, circSMARCA5 bond to its parent gene to inhibit SMARCA5 transcription^14^. Therefore, it is very important to study the regulation mechanisms of circRNAs in tumors. Whereas, the biology function and detailed molecular regulation mechanisms of circRNAs located in the nucleus in LSCC are still lacking.

In mammalian cells, as RNA modification, N6-methyladenosine (m6A) modification is the most prevalent and conserved^15^. It modified by “writers” containing METTL3, METTL14, and WTAP, eliminated by “erasers” including FTO and ALKBH5, recognized by “readers” with IGF2BP1/2/3 and YTHDC1/2/3^16^. M6A participated in a variety of biological processes involving RNA processing, nuclear transport, mRNA stabilization, translation and so on^17^. Previous studies had confirmed m6A exerts vital influence on cancers. In gastric cancer, METTL14 inhibited LHPP mRNA expression in a m6A-dependent manner to prevent the proliferation, invasion and metastasis of cancer cells. In LSCC, IGF2BP2, a m6A reader, identified and bound the Slug m6A site to support the stability of its mRNA^18^. It was recently discovered that m6A played an important role in the modification of circRNAs and regulated gene expression in tumors. In CRC, METTL3 induced circ1662 expression via marking m6A sites to promotes metastasis^19^. In HCC, circRNA-SORE was reduced by METTL3/14 in a m6A-dependent manner to maintain sorafenib resistance^20^.

In this study, a circRNA array was performed in three paired metastatic LSCC samples, and we found circMMP9 was significantly upregulated in LSCC tissues. High level of circMMP9 was strongly related to poor prognosis, low degree of pathological grading, high TNM stage and lymph node metastasis in LSCC. CircMMP9 stimulated proliferation and metastasis of LSCC cells in vitro and in vivo. Mechanically, we proved that IGF2BP2 stabilized circMMP9 in a m6A-dependent manner. Then circMMP9 recruited ETS1 to control TRIM59 transcription. Furthermore, we found that circMMP9-induced upregulation of TRIM59 contributed to the progression of LSCC via PI3K/AKT signal pathway.

## Results

### The expression and characteristics of circMMP9 in LSCC

**Figure 1:**
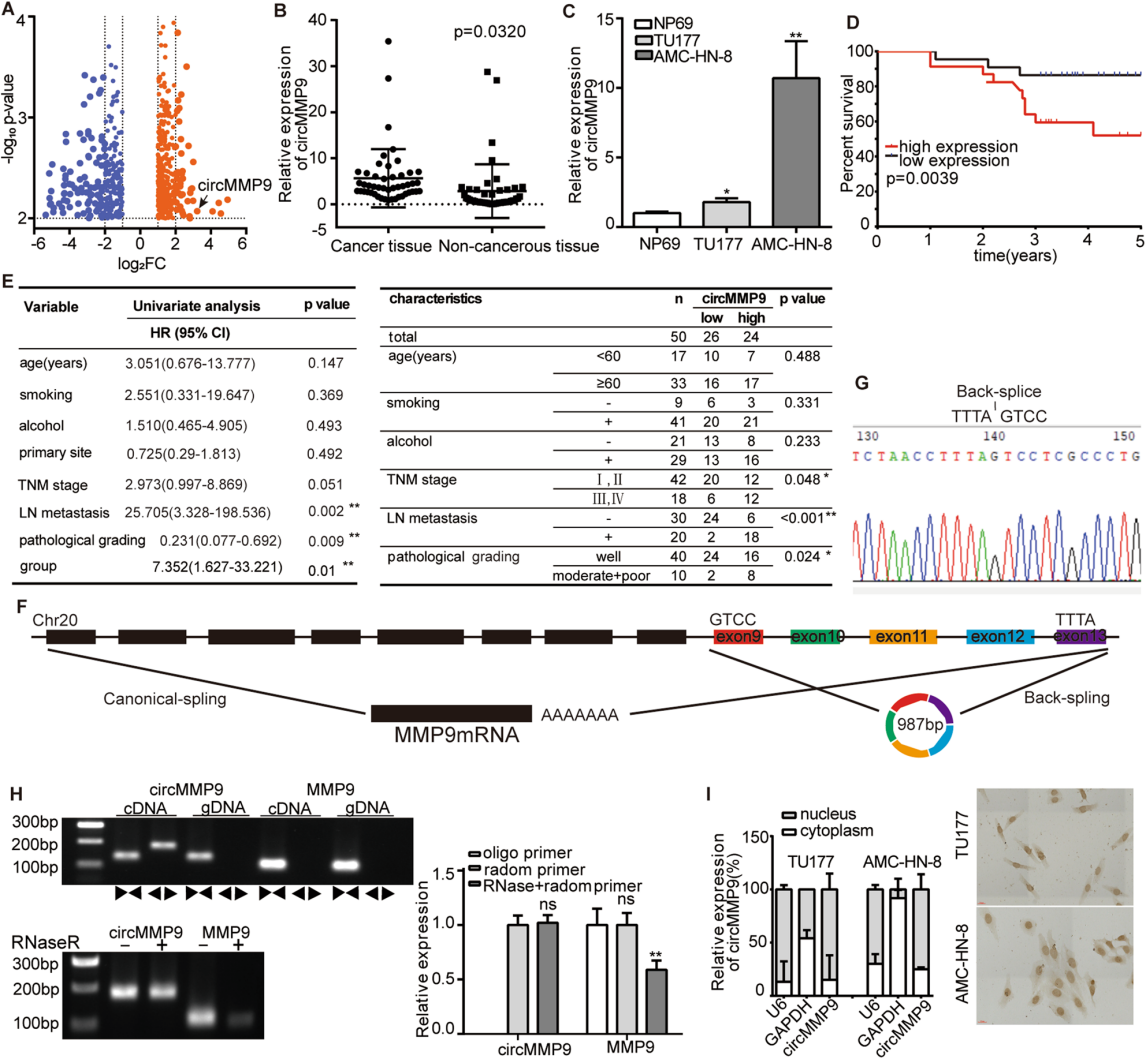
The expression and characteristics of circMMP9 in LSCC. (**A**) Volcanic map showing a total of 563 differentially expressed circRNAs in microarray (|log2fold change|> 1.0, *p* < 0.01), Orange dots represent 352 up-regulated, blue dots represent 211down-regulated circRNAs. (**B**) The qRT-PCR showing circMMP9 was upregulated in 50 LSCC tissues compared to adjacent non-cancerous tissues, (**C**) The qRT-PCR assay showed that circMMP9 was upregulated in TU177 and AMC-HN-8 compared to NP69. (**D**) Kaplan–Meier analysis showed high vs low expression of circMMP9 with OS in LSCC patients. (**E**) Univariate cox regression showing high circMMP9 expression, lymph node (LN) metastasis and low pathological grading was favorably correlated with poor OS (left); and multivariate analysis with clinicopathological factors showing high circMMP9 expression was closely correlated with low degree of pathological grading, high TNM stage and lymph node (LN) metastasis of LSCC (right). (**F**) CircMMP9 was formed following back-splicing of exon 9 to 13 of MMP9. (**G**) Sanger sequencing showed the junction site of circMMP9. (**H**) The result of PCR showed circMMP9 was amplified in cDNA but not gDNA using divergent primer but not convergent primers, and circMMP9 but not MMP9 could resist RNase R treatment. (**I**) The cellular distribution and in situ hybridization showed circMMP9 mainly located in the nucleus. Data represents mean ± S.D. from three independent experiments. Scale bars, 20 μm. ns, not significant, *p < 0.05; **p < 0.01.

Via analyzing the circRNA array with the criterion |log_2_FC|> 1.0, *p* < 0.01 (Supplementary Excel S1), a total of 563 differentially expressed circRNAs, involving 352 upregulated and 211 downregulated, were showed in the volcanic diagram (Fig. 1A). Then, we detected the top 5 circRNAs with the specific primers, and only hsa_circ_0060571(circMMP9) could be amplified in cDNA. Further, we verified circMMP9 expression in 50 paired samples of LSCC via qRT-PCR assay, and found a significant upregulation in tumor tissues relative to the contiguous non-cancerous tissues (Fig. 1B). Also, the above result was reconfirmed by TU177 and AMC-HN-8 cells compared to NP69 (Fig. 1C). Furthermore, Kaplan–Meier analysis revealed that high level of circMMP9 was unfavorable with overall survival (OS) in LSCC patients (*p* = 0.0039) (Fig. 1D). Moreover, high expression of circMMP9, lymph node (LN) metastasis and pathological grading were favorably correlated with OS by cox regression (Fig. 1E). Then multivariate analysis with clinicopathological factors showed that high expression of circMMP9 was favorably correlated with low degree of pathological grading, high TNM stage and lymph node metastasis (Fig. 1E). Via the above data, circMMP9 was essential to the poor prognosis of LSCC. By consulting circbase database, we found circMMP9 was a 987-nt circRNA generated from the exon 9 to 13 of the MMP9 (Fig. 1F). Then we used sanger sequencing to verify the junction site of circMMP9 (Fig. 1G). Next, to detect the trait of circMMP9, we amplified circular or linear MMP9 from cDNA and genomic DNA (gDNA) by convergent primers or divergent primers, then, added RNase R to the total RNA samples. The results proved that circMMP9 could be amplified by divergent primers in only cDNA but not in gDNA, and tolerate RNase R treatment (Fig. 1H). Furthermore, nuclear and cytoplasmic fractionation assay suggested that circMMP9 was mainly localized in nucleus, which was reconfirmed by in situ hybridization assay (Fig. 1I). The above results verified the expression and characteristics of circMMP9 in LSCC. According to the above-mentioned, the high expression of circMMP9, essential to the LSCC progression, was closely correlated with low degree of pathological grading, high TNM stage and lymph node metastasis in LSCC.

### The biological function of circMMP9 in vitro and in vivo

**Figure 2:**
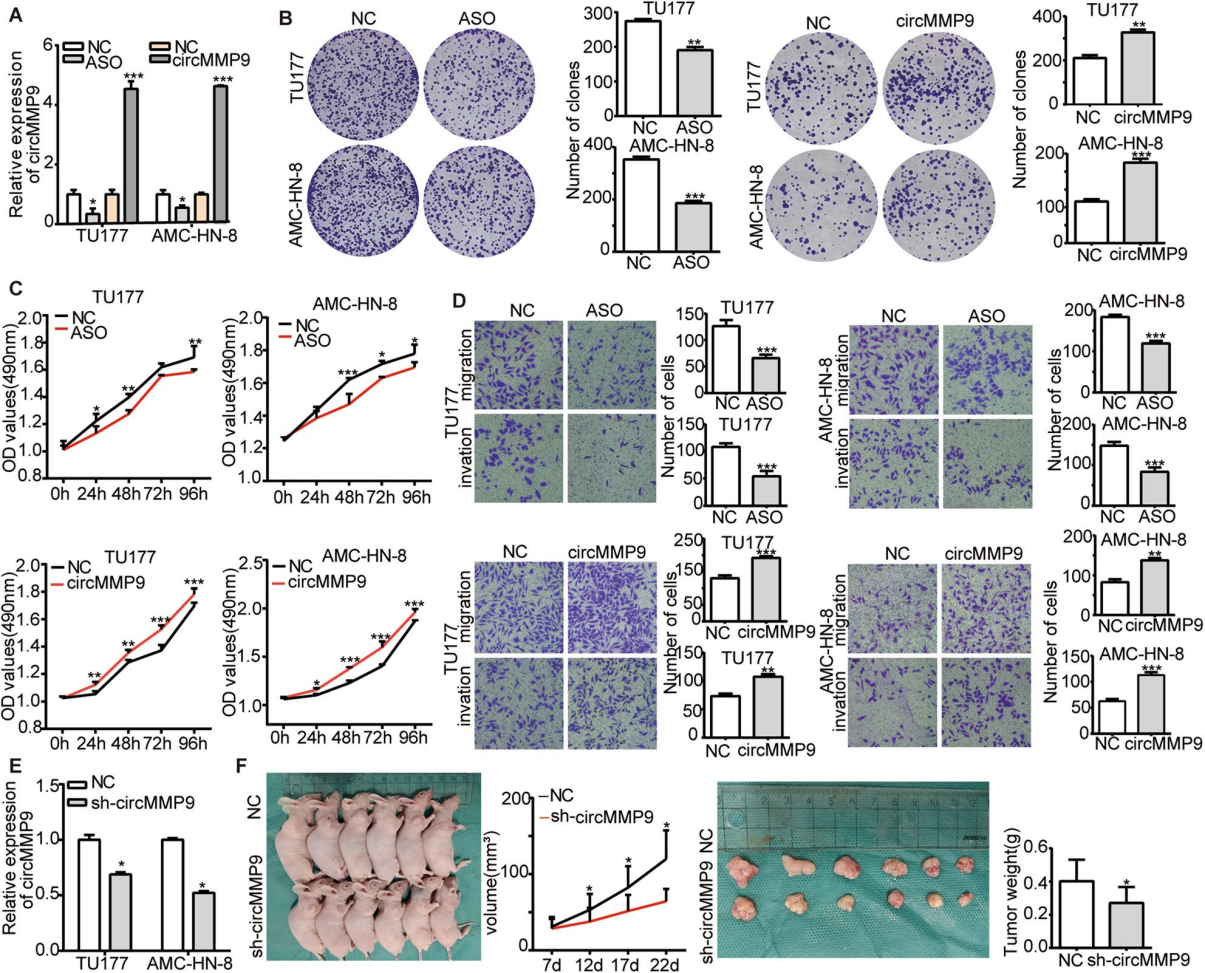
CircMMP9 promoted proliferation and invasion of LSCC cells in vitro and in vivo. (**A**) The qRT-PCR assay showed the knockdown efficiency of ASO of circMMP9, also the overexpression efficiency of transient transfection with pCD25-ciR plasmid (NC) and circMMP9 plasmid in TU177 and AMC-HN-8 cells. Cell biological function assays after knocking down or overexpressing circMMP9, (**B**) Colony formation assay showed that circMMP9 knockdown inhibited the proliferation ability of TU177 and AMC-HN-8 cells (left), however circMMP9 overexpression enhanced the proliferation ability of TU177 and AMC-HN-8 cells (right). (**C**) MTS assay showing the proliferation ability of TU177 and AMC-HN-8 cells was decreased in the absence of circMMP9 (up) and increased in the presence of circMMP9 (down). (**D**) Transwell assay showing the migration and invasion ability of TU177 and AMC-HN-8 cells was decreased in the absence of circMMP9 (up) and raised in the presence of circMMP9 (down). (**E**) The knockdown efficiency of pGreenPuro plasmid (NC) and circMMP9 stably knockdown lentivirus in TU177 and AMC-HN-8 cells. (**F**) xenograft tumor animal assay with control and circMMP9 knockdown lentivirus. Data represents mean ± S.D. from three independent experiments. *p < 0.05; **p < 0.01; ***p < 0.001.

We conducted cell biological function of circMMP9 in LSCC after the knockdown or overexpression efficiency of circMMP9 was evaluated via qRT-PCR (Fig. 2A). Via colony formation assay, we found that circMMP9 knockdown inhibited proliferation of LSCC cells, whereas circMMP9 overexpression played the opposite role (Fig. 2B). With MTS assay, we found that circMMP9 knockdown restrained proliferation, while circMMP9 overexpression facilitated proliferation of LSCC cells (Fig. 2C). Meanwhile, the transwell assay was conducted to explore the migration and invasion ability of circMMP9. The result verified that circMMP9 knockdown prevented migration and invasion, while circMMP9 overexpression promoted that of LSCC cells (Fig. 2D). Then we performed xenograft tumor animal assay after the efficiency of circMMP9 stably knockdown was tested (Fig. 2E). Consistent with in vitro assay, the result showed that circMMP9 stably knockdown inhibited proliferation of LSCC cells in vivo (Fig. 2F). All the above assays demonstrated that circMMP9 knockdown reduced the proliferation and metastasis of LSCC cells, however, circMMP9 overexpression had the opposite effect.

### CircMMP9 stabilized by IGF2BP2 in a m6A-dependent manner

**Figure 3:**
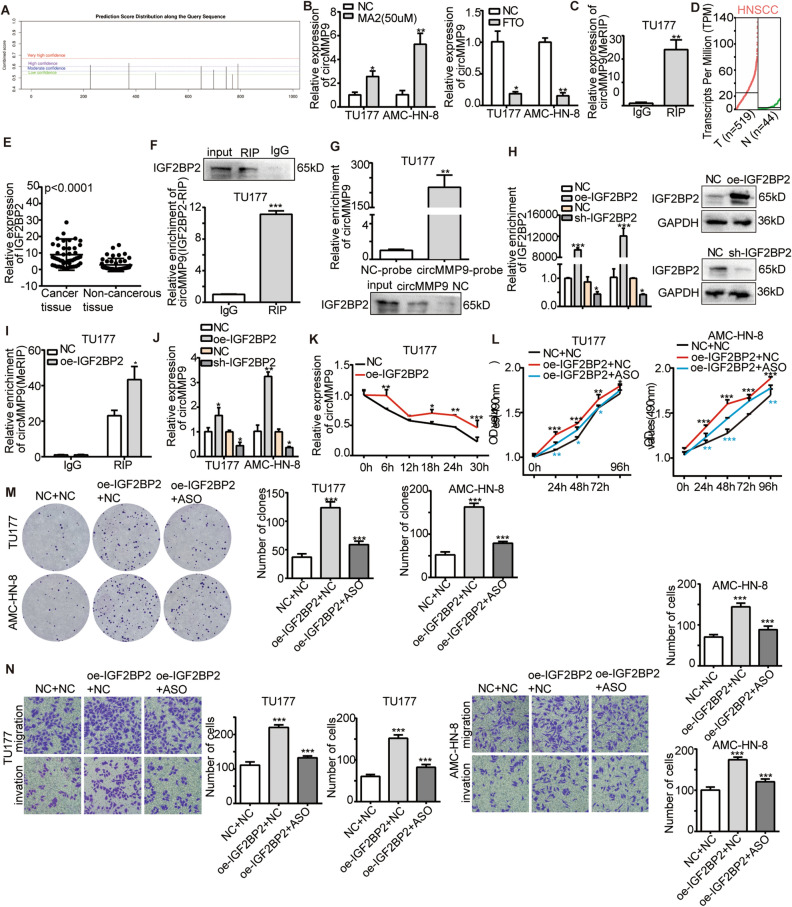
CircMMP9 was stabilized by IGF2BP2 in m6A-dependent manner. (**A**) SRAMP predicted 5 m6A sites on circMMP9 with a high degree of confidence. (**B**) The qRT-PCR showing circMMP9 decreased with the treatment of MA2(50 nM), increased with FTO overexpression in TU177, AMC-HN-8. (**C**) MeRIP-qPCR assays showing m6A antibody could bind more circMMP9 compared to the IgG group. (**D**) IGF2BP2 was upregulated in GEPIA on HNSCC. (**E**) The qRT-PCR assay showed that IGF2BP2 was upregulated in 50 LSCC tissues compared with adjacent non-cancerous tissues. (**F**) IGF2BP2-RIP-qPCR showing IGF2BP2 directly bound to circMMP9 compared to the IgG group. The original blots were presented in Supplementary Fig. 1A. (**G**) RNA-pulldown assay reconfirmed circMMP9 specifically bound to IGF2BP2. The original blots were presented in Supplementary Fig. 1B. (**H**) The qRT-PCR and WB assays showed the overexpression efficiency of transient transfection with pcDNA3.1 (NC) and IGF2BP2 plasmids, also the knockdown efficiency of transient transfection with pGenesil-1 (NC) and shRNA of IGF2BP2 in LSCC cells. The original blots were presented in Supplementary Fig. 1C,D. (**I**) MeRIP-qPCR showed that IGF2BP2 overexpression could enrich more circMMP9 than natural control. (**J**) The qRT-PCR showing circMMP9 was increased in the presence of IGF2BP2 and decreased in the absence of IGF2BP2 in TU177 and AMC-HN-8. (**K**) The actinomycin D treatment assay showing IGF2BP2 overexpression enhanced the stability of circMMP9 in TU177 cells treating with actinomycin D (4 μg/ml) for 6 h, 18 h, 24 h and 30 h. Cell biological function rescued assays, (**L**) MTS assay showed ASO of circMMP9 weakened the proliferation ability of TU177 and AMC-HN-8 cells caused by IGF2BP2 overexpression (black stars represent the comparison between NC + NC and oe-IGF2BP2 + NC; blue stars represent the comparison between oe-IGF2BP2 + NC and oe-IGF2BP2 + ASO). (**M**) Colony formation assay showed ASO of circMMP9 reduced the proliferation ability of TU177 and AMC-HN-8 cells induced by IGF2BP2 overexpression. (**N**) Transwell assay showed ASO of circMMP9 receded the migration and invasion ability of TU177 and AMC-HN-8 cells contributed by IGF2BP2 overexpression. Data represents mean ± S.D. from three independent experiments. ns, not significant, *p < 0.05; **p < 0.01; ***p < 0.001.

Previous studies had reported the regulation between m6A and circRNAs^21^. Therefore, we firstly predicted 5 m6A sites in circMMP9 with high degree of confidence via SRAMP (Fig. 3A). In order to verify the prediction, we treated TU177 and AMC-HN-8 cells with 50 mM MA2 (FTO inhibitor), and found that circMMP9 was increased in the presence of MA2, whereas circMMP9 was decreased with FTO overexpression (Fig. 3B). To reconfirm the above, we performed MeRIP assay and found that m6A antibody could bind more circMMP9 compared to the IgG group (Fig. 3C). All of the above results substantiated there was m6A modification on circMMP9. Moreover, we analyzed genes involved in m6A modification in GEPIA on HNSCC (head and neck squamous cell carcinoma), and found that only IGF2BP2 was upregulated (Fig. 3D). Then, via qRT-PCR assay, we reconfirmed IGF2BP2 was upregulated in 50 LSCC tissues compared to adjacent non-cancerous tissues (Fig. 3E). Previous studies had reported that IGF2BP2 was a vital m6A reader in identifying the m6A modification of circRNAs^22^. Thus, we assumed whether IGF2BP2 interacted with circMMP9 as a m6A reader. In order to testify the above assumption, we conducted IGF2BP2-RIP assay, and the result confirmed IGF2BP2 could directly bind to circMMP9 compared to the IgG group (Fig. 3F). To furtherly confirm this relationship, we performed RNA pulldown assay, the result reconfirmed the combination between circMMP9 and IGF2BP2 (Fig. 3G). Then we wondered if IGF2BP2 played an indispensable role in the m6A modification of circMMP9. Moreover, we proceeded MeRIP assay after the knockdown or overexpression efficiency of IGF2BP2 being evaluated via qRT-PCR and WB assays (Fig. 3H). The result verified m6A antibody enriched more circMMP9 in IGF2BP2 overexpression group than the natural control group (Fig. 3I). In addition, to investigate the impact of IGF2BP2 as a m6A reader on the expression of cirMMP9, we carried out qRT-PCR analysis and found that circMMP9 was decreased in the absence of IGF2BP2 and increased in the presence of IGF2BP2 (Fig. 3J). In light of the findings above, we assumed how m6A modification regulated circMMP9 expression in a m6A-dependent manner. Previous studies had reported that IGF2BP2 participated in the stabilization of RNA as a m6A reader^16^. Thereby, we initiated RNA stability assay under actinomycin D treatment with TU177 cells, and found that circMMP9 was more stable in IGF2BP2 overexpression group than natural control group (Fig. 3K). Moreover, we conducted cell functional assays including colony formation, MTS and transwell assay. The results suggested that circMMP9 knockdown reduced the proliferation, migration and invasion ability contributed by IGF2BP2 overexpression in LCSS cells (Fig. 3L–N). The above results revealed that circMMP9 was stabilized by IGF2BP2 in a m6A-dependent manner.

### CircMMP9 recruited ETS1 to promote TRIM59 transcription in LSCC cells

**Figure 4:**
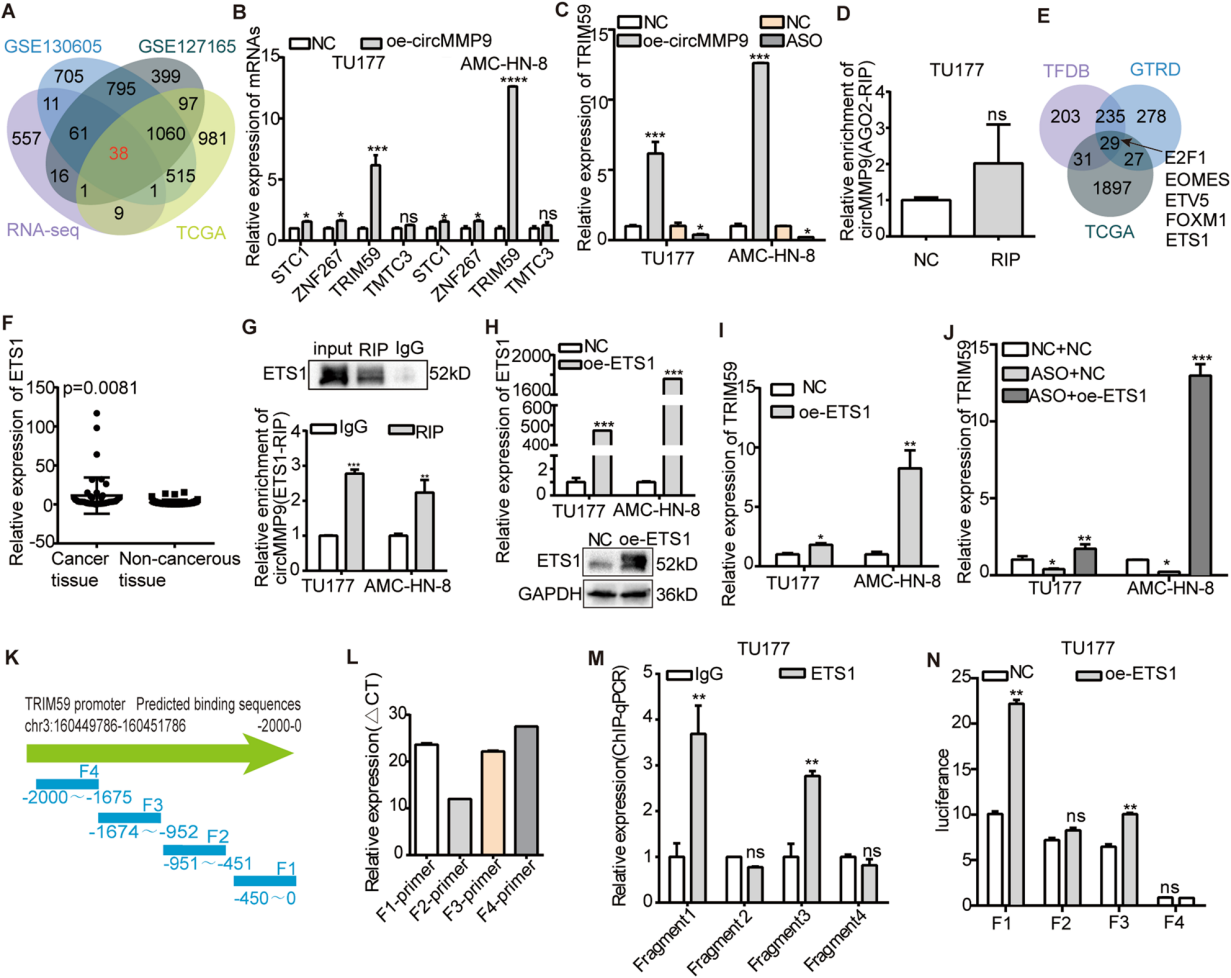
CircMMP9 promoted TRIM59 transcription through recruiting ETS1 in LSCC cells. (**A**) The Venn diagram showed the intersection result of RNA-seq (log_2_FC > 1, *p* < 0.0001), GSE130605 (log_2_FC > 0.5, *p* < 0.0001), GSE127165 (log_2_FC > 0.5, *p* < 0.0001) and The Cancer Genome Atlas (TCGA) datasets (log_2_FC > 0.5, *p* < 0.01) in LSCC, and there was a total of 38 mRNAs. (**B**) The qRT-PCR assay showed that the expression of TRIM59, STC1 and ZNF267 in the cross set was increased with circMMP9 overexpression. (**C**) The qRT-PCR assay showed TRIM59 was increased in the presence of circMMP9 and decreased in the absence of circMMP9 in TU177 and AMC-HN-8 cells. (**D**)AGO2-RIP-qPCR showing circMMP9 was unable to connect to AGO2 protein. (**E**) The transcription factors binding to the promoter region of TRIM59 (− 2000 bp to TSS) predicted via Human TFDB and GTRD databases, then crossing with The Cancer Genome Atlas (TCGA) datasets (log_2_FC > 0.5, *p* < 0.01) in LSCC, the Venn diagram listed the top 5 transcription factors. (**F**) ETS1 was upregulated in 50 LSCC tissues compared to paired non-cancerous specimens via qRT-PCR assay. (**G**) ETS1-RIP-qPCR showing ETS1 antibody could bind more circMMP9 compared to the IgG group. The original blots were presented in Supplementary Fig. 1E. (**H**) The qRT-qPCR and WB assays showed the overexpression efficiency of transient transfection with pcDNA3.1 (NC) and ETS1 plasmids. The original blots were presented in Supplementary Fig. 1F. (**I**) The qRT-qPCR assay showed the increase of TRIM59 in the present of ETS1. (**J**) The qRT-PCR showed that circMMP9 knockdown reduced TRIM59 expression caused by ETS1 overexpression in TU177 and AMC-HN-8 cells. (**K**) The promoter region of TRIM59 was divided into 4 fragments according to the predicted binding site. (**L**) The qRT-PCR assay verified primers of the 4 fragments. (**M**) ChIP-qPCR showed that ETS1 was enriched at the TRIM59 promoter fragments − 1647 to − 952 and − 451 to 0. (**N**) The dual-luciferase reporter assay revealed ETS1 enhanced the activity of TRIM59 promoter fragments − 1647 to − 952 and − 451 to 0 in TU177 cells. Data represents mean ± S.D. from three independent experiments. ns, not significant; *p < 0.05; **p < 0.01; ***p < 0.001.

To furtherly investigate the regulation mechanism of circMMP9 in LSCC, we performed and analyzed RNA-seq in vector and circMMP9 overexpression groups. Firstly, we selected the differentially expressed genes (DEGs) according to the criterion of log_2_FC > 1, *p* < 0.0001(Supplementary Excel S2). In order to strengthen our study's accuracy, we merged the mRNAs in RNA-seq with other LSCC datasets, including GSE130605, GSE127165, The Cancer Genome Atlas (TCGA) (Supplementary Excel S3), and found a total of 38 mRNAs listed in the Venn diagram (Fig. 4A). By searching the aforementioned genes in Genecard and associated publications in Pubmed, in addition, with the research of Jin et al. which verified that TRIM59 had diagnostic value in 15 solid tumors, including HNSC^23^, we ultimately chose TRIM59, STC1, ZNF267 and TMTC3 from the above 38 mRNAs as candidate target genes of circMMP9. Via qRT-PCR assay with circMMP9 overexpression in TU177 and AMC-HN-8 cells, we found that STC1, ZNF267 and TRIM59 were increased, with TRIM59 being the most upregulated (Fig. 4B). Meanwhile, that confirmed the reliability of RNA-seq. Then we reconfirmed TRIM59 was elevated in the presence of circMMP9 and reduced in the absence of circMMP9 via qRT-PCR assay (Fig. 4C). Moreover, we explored how circMMP9 controlled TRIM59 expression. First, since AGO2 was proved to attach to almost all miRNAs in human cells^24–26^, thus, it was possible to test whether circRNAs served as miRNA sponge via AGO2-RIP assay. Therefore, we tested whether circMMP9 served as “miRNA sponge” using AGO2-RIP. The result was unfavorable, indicating that circMMP9 failed to function as “miRNA sponge” (Fig. 4D). In view of circMMP9 localizing in the nucleus, we hypothesized if circMMP9 could recruit transcription factors to regulate TRIM59 transcription in LSCC. The Human TFDB and GTRD databases were then used to identify 264 transcription factors that were anticipated to be bound to the promoter region of TRIM59 (2000 bp to TSS). Given that the trend of TRIM59 in LSCC should be supported by the expression of transcription factors. Furthermore, we crossed the above 264 transcription factors with The Cancer Genome Atlas (TCGA) data on LSCC (with the criterion of log_2_FC > 1, *p* < 0.05), then listed the top 5 in the Venn diagram (Fig. 4E). According to our team's earlier research, ETS1 was elevated in LSCC and acted as a transcription factor^27^. As a result, ETS1 caught our attention with a significant possibility among these transcription factors. Also, we reconfirmed ETS1 was upregulated in 50 LSCC tissues compared to paired non-cancerous specimens via qRT-PCR assay (Fig. 4F). After that, we conducted ETS1-RIP assay to detect the relationship between circMMP9 and ETS1. The data revealed that ETS1 antibody could specially bind more circMMP9 compared to the IgG group (Fig. 4G). Next, we performed qRT-PCR assay to assess TRIM59 expression in the presence of ETS1 after the overexpression efficiency of ETS1 was detected via qRT-PCR and WB assays (Fig. 4H). The outcome demonstrated that ETS1 overexpression resulted in a considerable rise in TRIM59 (Fig. 4I). Next, we executed rescued assay, and the result showed ETS1 overexpression enhanced TRIM59 expression caused by circMMP9 knockdown (Fig. 4J). Consequently, the data above indicated that circMMP9 encouraged TRIM59 transcription by recruiting ETS1. Furtherly, we validated the above results via ChIP and dual-luciferase reporter assays. Firstly, we divided the promoter region of TRIM59 into 4 fragments and designed primers for each fragment according to the predicted binding site (Fig. 4K). Then we conducted ChIP assay after the primers of the 4 fragments had been tested (Fig. 4L). ChIP-qPCR showed that ETS1 enriched at the same site as the TRIM59 promoter fragments − 1647 to − 952 and − 451 to 0 (Fig. 4M). Furthermore, we proceeded dual-luciferase reporter after constructing the vectors of the above 4 fragments respectively. The result revealed ETS1 overexpression enhanced TRIM59 promoter activity of the − 1647 to − 952 and − 451 to 0 (Fig. 4N). The above data reconfirmed that ETS1 promoted the transcription of TRIM59. By overlapping the results of RNA-seq, ChIP and dual-luciferase reporter assay, we verified that TRIM59 was a target gene for both circMMP9 and ETS1 in LSCC cells. In brief, circMMP9 could recruit ETS1 and stimulate TRIM59 transcription in LSCC.

### TRIM59 promoted LSCC cells metastasis through PI3K-AKT pathway

**Figure 5:**
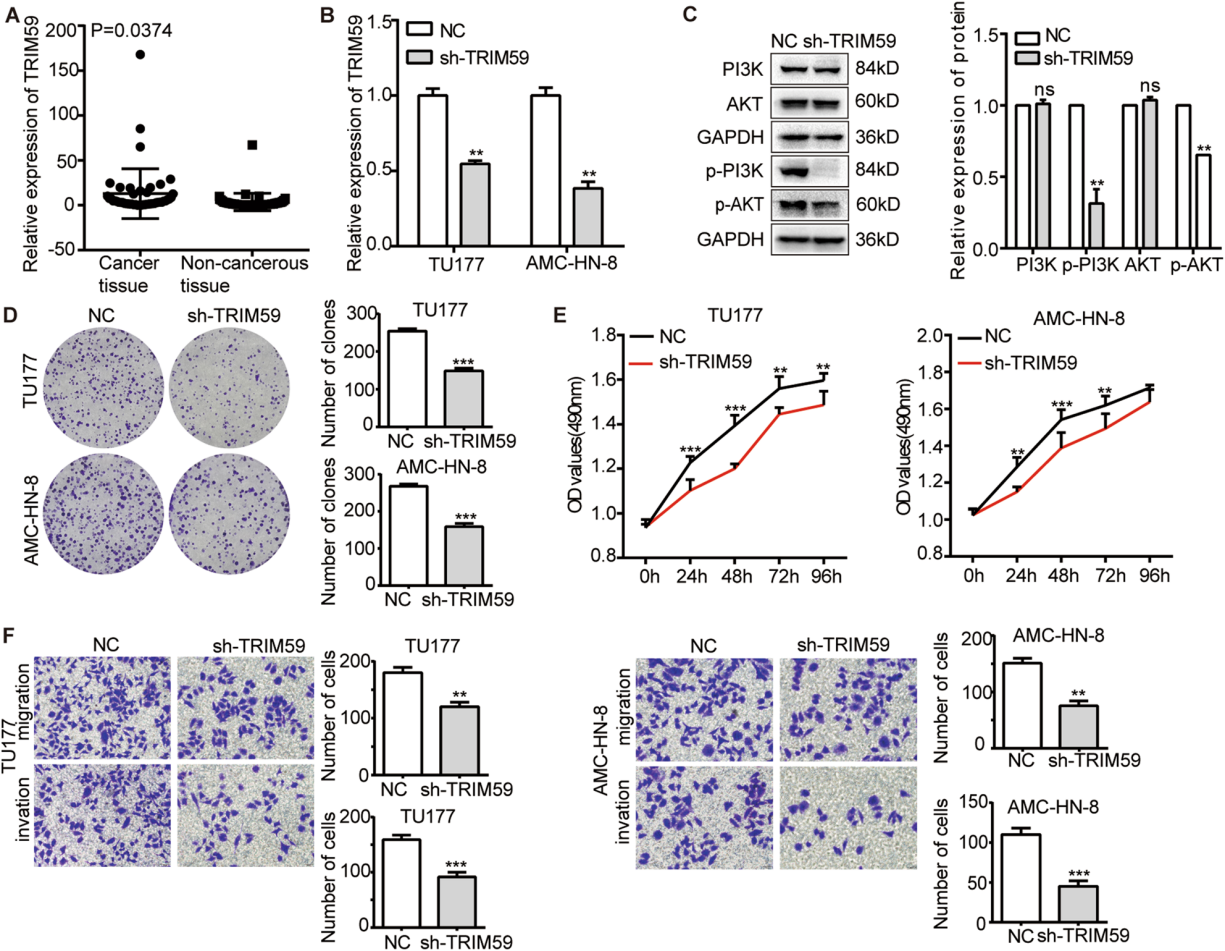
TRIM59 promoted LSCC cells metastasis through PI3K-AKT pathway. (**A**) The qRT-PCR assay showed that TRIM59 was upregulated in 50 LSCC tissues compared with adjacent noncancerous tissues. (**B**) The qRT-PCR assay showed the knockdown efficiency of transient transfection with pGenesil-1 (NC) and shRNA of TRIM59 in LSCC cells. (**C**) The WB analysis demonstrated the reduction in phosphorylation level of PI3K and AKT with TRIM59 knockdown in LSCC cells. The original blots were presented in Supplementary Fig. 1G. Cell biological function assays, (**D**) Colony formation assay showed TRIM59 knockdown inhibited the proliferation ability of TU177 and AMC-HN-8 cells. (**E**) MTS assay showed TRIM59 knockdown restrained the proliferation ability of TU177 and AMC-HN-8 cells. (**F**) Transwell assays showed TRIM59 knockdown attenuated the migration and invasion ability of TU177 and AMC-HN-8 cells. Data represents mean ± S.D. from three independent experiments. ns, not significant; *p < 0.05; **p < 0.01; ***p < 0.001.

Previous studies had revealed that TRIM59 was an essential factor of tumor progression^28,29^. Also, according to Li et al., TRIM59 triggered the PI3K-AKT pathway to hasten the progression of pancreatic cancer^30^. Via qRT-PCR assay, we found TRIM59 was upregulated in 50 LSCC tissues compared to matching non-cancerous specimens (Fig. 5A). To furtherly investigate the role of TRIM59 in LSCC progression, we manipulated the expression of TRIM59 in LSCC cells, and the knockdown efficiency was confirmed by qRT-PCR assay (Fig. 5B). Then, we examined the markers of PI3K-AKT pathway (PI3K, p-PI3K, AKT and p-AKT) in the absence of TRIM59. The WB analysis demonstrated the phosphorylation level of PI3K and AKT decreased with TRIM59 knockdown in LSCC cells (Fig. 5C). Additionally, we proceeded cell biology functional assays including colony formation, MTS and transwell assay in LSCC cells. The findings showed that TRIM59 knockdown greatly reduced the capacity of LSCC cells to proliferate, migrate, and invade (Fig. 5D–F). In conclusion, TRIM59 contributed to the progression of LSCC by triggering the PI3K-AKT signal pathway.

### CircMMP9 accelerated LSCC progression via TRIM59

**Figure 6:**
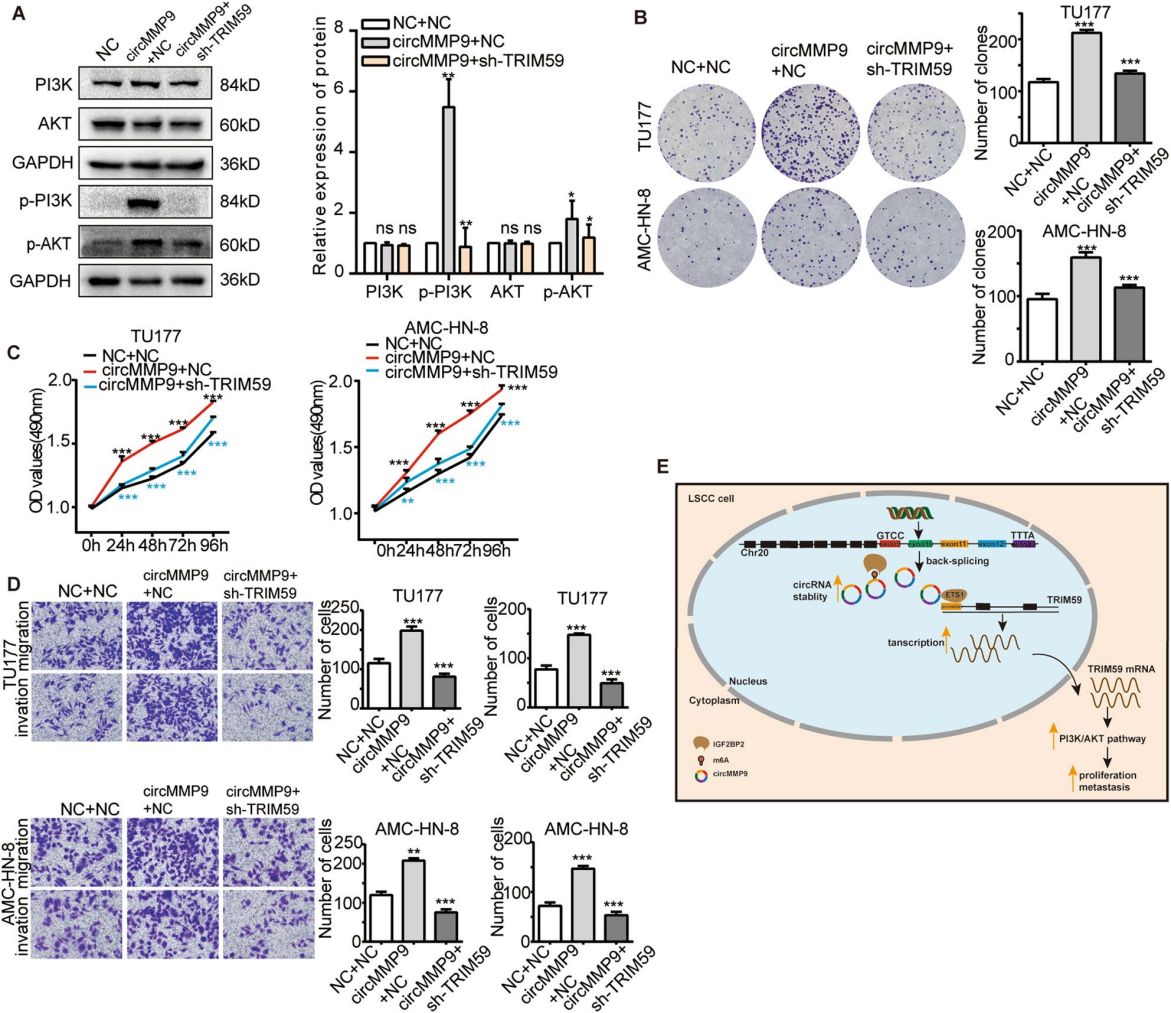
CircMMP9 enhanced LSCC progression via TRIM59. (**A**) The WB analysis showed that the increased phosphorylation level of PI3K and AKT caused by circMMP9 overexpression could be rescued by TRIM59 knocking down. The original blots were presented in Supplementary Fig. 1H. Cell biological function rescued assays (**B**) Colony formation assay showed that TRIM59 knockdown weakened the proliferation ability of TU177 and AMC-HN-8 cells caused by circMMP9 overexpression. (**C**) MTS assay showed TRIM59 knockdown reduced the proliferation ability of TU177 and AMC-HN-8 cells induced by circMMP9 overexpression (black stars represent the comparison between NC + NC and circMMP9 + NC; blue stars represent the comparison between circMMP9 + NC and circMMP9 + sh-TRIM59). (**D**)Transwell assays showed TRIM59 knockdown weakened the migration and invasion ability of TU177 and AMC-HN-8 cells contributed by circMMP9 overexpression. (**E**) The mechanism diagram of circMMP9 promoted LSCC progression. Data represents mean ± S.D. from three independent experiments. ns, not significant; *p < 0.05; **p < 0.01; ***p < 0.001.

In view of the fact that circMMP9 regulated the transcription of TRIM59, we furtherly investigated if circMMP9 could regulate the PI3K-AKT pathway in LSCC. Via WB analysis we discovered that TRIM59 knockdown reduced the expression of p-PI3K and p-AKT caused by circMMP9 overexpression (Fig. 6A). Then we carried on cell biology functional rescued assays, the results verified that TRIM59 knockdown could reverse the promoting impact contributed by circMMP9 overexpression in LSCC cells (Fig. 6B–D). These results demonstrated that circMMP9 facilitated LSCC progression by way of TRIM59 (Fig. 6E).

## Discussion

As novel non-coding RNA, circRNAs are single-stranded, covalently closed RNA molecules with tissue specificity and expression stability^31^. Numerous circRNAs had been identified in cancers and played important biological functions. For instance, circACTN4 promoted liver cancer progression^32^. CircumNDUFB2 halted lung cancer from progressing by triggering anti-tumor immunity^33^. In contrast, the role of the circRNAs in LSCC has been seldom involved. In the current study, we profiled circRNA microarray in three pairs of metastatic LSCC and adjacent non-cancerous tissues, then identified that circMMP9 was upregulated in LSCC tissues. CircMMP9 was formed by reverse cutting of exon 9–13 of MMP9. Then we discovered 3 circRNAs formed by MMP9 via circbase database, including hsa_circ_0001161, hsa_circ_0001162 and hsa_circ_0060571(circMMP9). Previous research had verified hsa_circ_0001162 upregulated in glioblastoma multiforme and Osteosarcoma promoted tumor progression^34,35^. However, at present, there is no research on circMMP9 in tumor. In our study, we confirmed circMMP9 upregulated could act as a promoter in proliferation, migration and invasion of LSCC cells both in vitro and in vivo.

Thus an increasing body of evidences verified that circRNAs were associated with clinicopathologic features and prognosis in tumor patients^36,37^. In our study, we found higher circMMP9 expression was significantly correlated with lower survival, lower degree of pathological grading, higher TNM stage and lymph node metastasis of LSCC than lower expression. More important, accumulating research suggested circRNAs regulated tumor progression as molecular markers through multiple mechanisms, including function as sponge for miRNAs, scaffolds for proteins, templates for translation, and regulators of mRNA translation and stability^38,39^. In liver tumor, circRNA cia-MAF recruited the TIP60 complex to promote the transcription of MAFF^40^. In cervical cancer, circCCDC134 recruited p65 in the nucleus stimulating HIF1A transcription and facilitating tumor growth and metastasis. In this research, we detected the role of the endo-nucleus circMMP9 in LSCC. As a key transcription factor, ETS1 played an important role in a variety of diseases^41,42^. For example, ETS1 exerted vital influence on tumors including breast cancer^43^. Also, ETS1 was identified as a transcription factor promoting LSCC progression^27^. In this study, we found circMMP9 could recruit ETS1 to enhance the transcription of downstream target gene, which enriched the research mechanism of circRNAs in the nucleus.

TRIM59 is a key member of the TRIM family and acts a vital role in cancers. Han et al. reported TRIM59 played an important role in autophagy regulation in NSCLC^44^. Moreover, there were growing evidences verified that TRIM59 activated PI3K-AKT signal pathway in tumors. In colorectal cancer, TRIM59 promoted metastasis via the PI3K/AKT pathway^45^. And in breast cancer, TRIM59 was correlated with poor prognosis and contributed to progression through AKT signaling pathway^46^. However, the mechanism of TRIM59 in LSCC has not been discussed. In our study, we found that circMMP9 recruited ETS1 to enhance TRIM59 transcription. Furthermore, TRIM59 could promote LSCC progression via activating PI3K/AKT signal pathway.

The m6A methylation, contained METTL3/14, WTAP as “writers”, FTO, ALKBH5 as “erasers”, and IGF2BP1/2/3, YTHDC1/2/3 as “readers”^47^. Functionally, m6A exerted vital influence on cancers. Shi et al. reported that METTL14 regulated gene expression in gastrointestinal cancer^48^. Weng et al. found IGF2BP2 functioned as a typical m6A reader in AML progression^49^. In this study, we found IGF2BP2 also functioned as a m6A reader. Lately, it had been shown m6A modification on circRNAs acted crucial function in cancers via different mechanisms which included RNA splicing, localization, stabilization, translation and degradation^21,50,51^. In CRC, circNSUN2 promoted colorectal liver metastasis via facilitating cytoplasmic export by YTHDC1 in m6A-dependent manner and stabilizing HMGA2^52^. In NSCLC, circIGF2BP3 mediated by METTL3 in m6A-dependent manner could cause immune escape^53^. In our data, we discovered circMMP9 was stabilized by IGF2BP2 in a m6A-dependent manner.

To summarize, in our data, circMMP9 exhibited a significant role in LSCC progression. High circMMP9 level was closely correlated with poor prognosis, low degree of pathological grading, high TNM stage and lymph node metastasis in LSCC patients. Mechanistically, circMMP9 was stabilized by IGF2BP2 in a m6A-dependent manner, and circMMP9 recruited ETS1 to promote TRIM59 transcription. Moreover, the circMMP9-dependent upregulation of TRIM59 activated the PI3K/AKT signal pathway and promoted LSCC progression. Our finding suggested that circMMP9 might play an oncogenic role in the progression of LSCC and serve as a new diagnostic and prognostic marker, as well as a therapeutical target for progression of LSCC patients.

## Conclusions

In conclusion, we confirmed that circMMP9 promoted LSCC progression in vitro and in vivo. Also, high circMMP9 was unfavorable with prognosis in LSCC patients. IGF2BP2-m6A-circMMP9 axis recruited ETS1 to promote TRIM59 transcription in LSCC. Moreover, the circMMP9-induced upregulation of TRIM59 activated the PI3K/AKT signal pathway and promoted LSCC progression.

## Methods

### Clinical sample

50 pairs of LSCC samples were obtained from the Second Hospital of Hebei Medical University with informed consent. All treatments were approved by the Ethics Committee of Second Hospital of Hebei Medical University in this study. Clinicopathological features of the patients were presented in Supplementary Table S1.

### Cell line culture

Human normal nasopharyngeal cells (NP69), 293 T, TU177 and AMC-HN-8 cells were purchased from Beijing Beina Chuanglian Institute of Biotechnology (Beijing, China). With the protocols of our team's previous research, 293 T, TU177 and AMC-HN-8 cells were cultured^54^. And NP69 cells were cultured at 37˚C with 5% CO2 in Keratinocyte-SFM medium (Gibco; Thermo Fisher Scientific, Inc.) mixed with 10% fetal bovine serum.

### CircRNA microarray

CircRNA microarray was completed by OE Biotechnology Co., Ltd., (Shanghai, China). Total RNA was obtained using mirVana™ RNA Isolation Kit (AM1561). Agilent Bioanalyzer 2100 (Agilent Technologies) was used to evaluate RNA integrity and the NanoDrop ND-2000 (Thermo Scientific) was used to check RNA quality. Then circRNAs were hybridized by the Agilent Human ceRNA Microarray 2019 (4 × 180 k). In accordance with the manufacturer’s recommended procedures, sample labeling, microarray hybridization and washing were all carried out sequentially. Furthermore, the tagged circRNAs were hybridized onto the microarray. After washing, the Agilent Scanner G2505C (Agilent Technologies) was used to scan the arrays. And raw data was collected by Feature Extraction software (version10.7.1.1, Agilent Technologies). Then the original analysis was processed by Genespring software (version 14.8, Agilent Technologies). Differentially expressed circRNAs were screened by the criterion of |log_2_fold change|> 1 and *p* value < 0.05.

### RNA sequencing

Majorbio Bio-pharm Technology Co.,Ltd (Shanghai, China) performed the RNA sequencing process. According the manufacturer's guidelines, total RNA from TU177 cells transfected with the vector and circMMP9 overexpression was extracted using TRIzol (Invitrogen, Carlsbad, CA, USA), and genomic DNA was eliminated utilising DNase I (TaKara). Then, RNA quality was evaluated with an Agilent 2100 Bioanalyser and quantified via a NanoDrop Technologies ND-2000. With 1 μg of total RNA, an RNA-seq transcriptome library was established by the TruSeqTM RNA sample preparation Kit from Illumina (San Diego, CA). Next, combining with oligo (dT) beads and the polyA selection process, messenger RNA (mRNA) was extracted and then fragmented. Using random hexamer primers from Illumina and a SuperScript double-stranded cDNA synthesis kit from Invitrogen in California, double-stranded cDNA was created. Paired-end RNA-seq sequencing library was sequenced with the Illumina HiSeq xten after being quantified by TBS380. The raw paired end reads were trimmed and quality-check by SeqPrep and Sickle with default parameters. Then, by employing the DEGseq, differential expression analysis was carried out.

### Plasmid or oligonucleotide construction and transfection

The circRNA overexpression plasmid was bought from Geneseed Biotech (Guangzhou, China). IGF2BP2, ETS1 and the knockdown lentivirus plasmids were purchased from Youbio Biological Technology (Changsha, China). We designed shRNA (short hairpin RNA) to knockdown the expression of circMMP9, IGF2BP2 and TRIM59. ASO (antisense oligonucleotides) was designed and synthesized by RiboBio (Guangzhou, China) to specifically knock down circMMP9. The linear fragment of circMMP9 together with the flanking sequence which induced circRNA was subcloned into EcoRI and BamHI sites of pCD25-ciR. The plasmids and shRNA were transfected by Hieff Trans Liposomal Transfection Reagent (Yeasen). ASO and lentivirus plasmid were transfected by Lipofectamine 2000 (Invitrogen, Carlsbad, CA, USA) according to the manufacturers’ instruction. The sh-circMMP9 sequence was cloned into pGreenPuro. Then we transfected pGreenPuro, pSBH155 and pSBH156 into 293 T cells to obtain lentiviral supernatant. After 48 h, we used density gradient centrifugation to collect and concentrate viruses, then frozen them at − 80 °C for follow-up research. The sequences of ASO, negative control (ASO-NC) and shRNA in this study were listed in the Supplementary Table S2.

### RNA extraction, RNase R treatment, actinomycin D and quantitative real-time PCR assays

Total RNA was extracted from LSCC tissues or cells using TRIzol reagent (Invitrogen, CA, USA). Cytoplasmic and nuclear fractions were isolated using NE-PER Nuclear and Cytoplasmic Extraction Reagents (ThermoFisher Scientific, USA) from TU177 and AMC-HN-8 cells. Total RNA was added with or without 3U/ug RNase R (Epicentre, WI, USA) and incubated for 5 min at 37 °C. Actinomycin D was used for the treatment of TU177 cells at 0, 6, 12, 18 and 24 h before RNA extraction to detect the expression of circMMP9. First-strand cDNA was synthesized using HiFiScript cDNA Synthesis Kit (Roche). Real-time quantitative PCR (qRT-PCR) analysis was conducted with Go Taq qPCR Master Mix (Promega Scientific, USA) according to the manufacturers’ instructions. The relative expression of RNAs were calculated using 2^−ΔΔct^. The specific primers were listed in the Supplementary Table S2.

### Western blot assay

Western blot (WB) assay was conducted as follows: we added RIPA lysis buffer with PMSF (Solarbio, China) and protease inhibitor cocktail (Promega, USA), to either TU177 or AMC-HN-8 cells before protein was extracted. Protein concentration was detected by the BCA Protein Assay Kit (Generay, China). Protein lysates were heated at 95 °C for 5 min with SDS-PAGE loading buffer (Solarbio, China). Then 20ug protein was separated by SDS-PAGE. According to the molecular weight of the antibodies, the SDS-PAGE were cut prior to hybridization with antibodies. Next the SDS-PAGE which had been cut were transferred onto PVDF membranes (Bio-Rad, USA). 5% skim milk (BD, USA) was used for blocking membranes at room temperature for 2 h. Primary antibodies were added to the blots then incubated at 4 °C overnight. Then the blots were incubated with secondary antibodies at room temperature for 2 h. ChemiDocTM XRS + (Bio-Rad, USA) and enhanced chemiluminescence (ECL) detection reagents (vazyme, China) were used to detect the band. The information of primary antibodies was listed in the Supplementary Table S3.

### In situ hybridization

Biotin-labeled probes of circMMP9 was designed and synthesized by RiboBio (Guangzhou, China). The oligonucleotide sequence was listed in Supplementary Table S2. We cultured TU177 and AMC-HN-8 cells on the cover glasses. Then cells were fixed with 4% paraformaldehyde for 30 min, before permeabilized by 0.25% Triton-X100 for 10 min. Next, 3% H_2_O_2_ was added to cells for 10 min to block endogenous peroxidases. Then biotin-labeled probes of circMMP9 was used to hybridize at 37 °C overnight. After that, cells were rinsed in SSC buffer at 42 °C. Then cells were incubated with a homologous HRP-labeled Streptavidin (Sangon Biotech, Shanghai, China) for 37 °C, 30 min and stained using DAB staining kit. The result was detect using the microscope (OLYMPUS, Japan).

### Colony formation assay

TU177 and AMC-HN-8 cells were resuspended in culture medium with 10% FBS. 2 × 10^3^ cells were seeded per well in 6-well plates, then cells were incubated at 37 °C for 11 days, after being fixed the colonies were stained with 1% crystal violet prior to more than 50 cells were counted.

### MTS assay

The viability of LSCC cells was measured by MTS Assay Kit (Sigma, St. Louis, MO, USA). 2 × 10^3^ cells were seeded in 96-well plates, then 20 μl MTS Assay Kit was added to each well and incubated for 2 h at 37 °C. The absorbance was detected using Spark® multimode microplate reader (Mod: SPARK 10 M, TECAN, Switzerland) at a wavelength of 490 nm.

### Transwell assay

TU177 and AMC-HN-8 cells were suspended in culture medium without FBS. After 48 h transfected with specific oligonucleotides or plasmids. Then, 1 × 10^5^ cells were seeded into the upper chamber, while the lower chamber was plated into a 650ul medium with 10% FBS. then incubated at 37 °C for 24 h, the migrated cells were fixed and stained crystal violet (1%) at room temperature. Finally, the filter membrane was photographed and counted in three random fields.

### Animal model in vivo

We carried on xenografts assay using 4-week-old female BALB/c nude mice, and they were fed in specific pathogen-free conditions. HeBei Medical University Animal Care and Use Committee approved all procedures of this assay. 5 × 10^6^ (200ul) TU177 cells stably transfected with lentiviruses carrying sh-circMMP9/sh-NC were subcutaneously inoculated. We measured and calculated the tumor volume with the formula (length × width^2^/2) per 7 days. After 28 days, the mice were anesthetized by intraperitoneal injection of pentobarbital sodium (150 mg/kg), then the mice were quickly sacrificed by cervical dislocation. The tumors were collected for further study.

### RNA immunoprecipitation (RIP)

PureBinding®RNA Immunoprecipitation Kit (Geneseed Bio, Guangzhou, China) was used to perform the RIP assay according to the manufactures’ guidelines. Briefly, 2 × 10^7^ LSCC cells were harvested and lysed in RIP lysis buffer. After centrifugation at 4 °C, the supernatant was incubated with specific antibodies and negative control IgG at room temperature. The beads-antibody complex was then washed and incubated with Proteinase K. The immunoprecipitated RNA was purified and detected by RT-qPCR.

### RNA pulldown assay

CircMMP9 pulldown assay was performed with a CHIRP kit (Bersinbio) according to the manufacturer’s instructions. Biotin-labeled circMMP9 probe or NC probe was synthesized by RiboBio (Guangzhou, China), and the sequence was complemented to the junction site of the circMMP9 (listed in the Supplementary Table S2). 4 × 10^7^ LSCC cells were lysed in 500ul IP buffer, and incubated with circMMP9 probe for 2 h at room temperature. Then, 20ul Streptavidin magnetic beads were added to the reaction for another an hour of incubation. Finally, the beads were washed five times, followed by western blot detection.

### Chromatin immunoprecipitation assay (ChIP)

ChIP assay was performed using the Magna ChIP A/G Kit (Millipore, Germany) according to the manufacturers’ instructions. Briefly, TU177 cells were cross-linked with 1% formaldehyde and quenched with glycine. After being washed with four times and de-crosslinked, the precipitated DNA was amplified and detected by qRT-PCR. The specific ChIP primers were listed in the Supplementary Table S2. The enrichment of ETS1 on the promoter region of TRIM59 in TU177 was determined by ChIP-qPCR assay. According to the manufacturer’s protocol, TU177 cells were cultured in 15 cm culture dish. They were fixed in 1% formaldehyde. Then, the cells were lysed and sonicated to the ~ 200–500 bp DNA fragments in order to create appropriately sized chromatin fragments, ETS1 antibody and IgG antibody were used to precipitate the specific DNA fragment. After purification, ChIP-derived DNA was used as templet for qRT-PCR analysis, and the enrichment was relative to input. The primers were shown in Supplementary Table S1.

### Luciferase reporter assay

For the TRIM59 promoter-luciferase reporter assay, we divided the promoter region of TRIM59 (− 2000 bp to TSS) into 4 sections according to the ETS1 binding sites. Then we constructed the luciferase reporter vectors containing the above 4 sections. All primers were listed in Supplementary Table S2. DNA was used as PCR templates. The vectors with correct fragments verified by sequencing were digested with Nhel and HindIII (Thermo Scientific, USA). DNA fragments were ligated respectively to the pGL3-basic luciferase vectors. TU177 cells were transfected with luciferase reporter plasmids. The control group was co-transfected with 1500 ng plasmids, 50 ng pRL-TK and 1500 ng pCDNA3.1, and the other group was co-transfected with 1500 ng plasmids, 50 ng pRL-TK and 1500 ng ETS1, then incubated at 37 °C for 48 h. The luciferase activity was measured using the Dual-Luciferase®Reporter Assay System (Promega, USA) according to the manufacturer’s instructions. Promoter activities were expressed as the ratio of Firefly luciferase to Renilla luciferase activity.

### Statistical analysis

The results were presented as the means ± SD. All the data was analyzed by SPSS 25.0 (Chicago, USA). The figures were plotted by GraphPad Prism 6 (GraphPad Software, Inc., CA). Kaplan–Meier plot was used for survival analysis. Differences between groups were evaluate by the student’s t test or the unpaired two sides t test. The *p* value < 0.05 was considered to be statistically significant.

### Ethical approval and consent to participate

All treatments in this study were approved by the Ethics Committee of The Second Hospital of Hebei Medical University. Statement We confirm this study is reported in accordance with ARRIVE guidelines.

### Supplementary Information

Supplementary Information 1.

Supplementary Information 2.

Supplementary Information 3.

Supplementary Information 4.

Supplementary Information 5.

Supplementary Table S1.

Supplementary Table S2.

Supplementary Table S3.

## Data Availability

Data and materials used and/or analyzed during the current study are available within the manuscript and its supplementary information files.

## References

[1] Siegel RL (2017). Cancer Statistics, 2017. CA Cancer J, Clin..

[2] Siegel RL (2015). Cancer statistics, 2015. CA Cancer J. Clin..

[3] Cramer JD (2019). The changing therapeutic landscape of head and neck cancer. Nat. Rev. Clin. Oncol..

[4] Carlisle JW (2020). An update on the immune landscape in lung and head and neck cancers. CA Cancer J. Clin..

[5] Johnson DE (2020). Head and neck squamous cell carcinoma. J. Nat. Rev. Dis. Primers.

[6] Memczak S (2013). Circular RNAs are a large class of animal RNAs with regulatory potency. J. Nat..

[7] Tao M (2021). CircRNAs and their regulatory roles in cancers. J. Mol. Med..

[8] Bu J (2022). The circRNA circADAMTS6 promotes progression of ESCC and correlates with prognosis. J. Sci. Rep..

[9] Wang S (2021). Circular RNAs in body fluids as cancer biomarkers: The new frontier of liquid biopsies. J. Mol. Cancer.

[10] Li C (2019). Circular RNAs: Pivotal molecular regulators and novel diagnostic and prognostic biomarkers in non-small cell lung cancer. J. Cancer Res. Clin. Oncol..

[11] Chen LL (2016). The biogenesis and emerging roles of circular RNAs. J. Nat. Rev. Mol. Cell Biol..

[12] Li Z (2015). Exon-intron circular RNAs regulate transcription in the nucleus. J. Nat. Struct. Mol. Biol..

[13] Jie M (2020). CircMRPS35 suppresses gastric cancer progression via recruiting KAT7 to govern histone modification. J. Mol. Cancer.

[14] Xu X (2020). CircRNA inhibits DNA damage repair by interacting with host gene. J. Mol. Cancer.

[15] Barbieri I (2020). Role of RNA modifications in cancer. J. Nat. Rev. Cancer.

[16] Yang Y (2018). Dynamic transcriptomic m(6)A decoration: Writers, erasers, readers and functions in RNA metabolism. J. Cell Res..

[17] Frye M (2018). RNA modifications modulate gene expression during development. J. Sci..

[18] Yu D (2022). RNA N6-methyladenosine reader IGF2BP2 promotes lymphatic metastasis and epithelial-mesenchymal transition of head and neck squamous carcinoma cells via stabilizing slug mRNA in an m6A-dependent manner. J. Exp. Clin. Cancer Res..

[19] Chen C (2021). N6-methyladenosine-induced circ1662 promotes metastasis of colorectal cancer by accelerating YAP1 nuclear localization. J. Theranostics.

[20] Xu J (2020). N(6)-methyladenosine-modified CircRNA-SORE sustains sorafenib resistance in hepatocellular carcinoma by regulating beta-catenin signaling. J. Mol. Cancer.

[21] Wang X (2021). Crosstalk between N6-methyladenosine modification and circular RNAs: Current understanding and future directions. J. Mol. Cancer.

[22] Liu Z (2022). N6-methyladenosine-modified circular RNA QSOX1 promotes colorectal cancer resistance to anti-CTLA-4 therapy through induction of intratumoral regulatory T cells. Drug Resist. Updates.

[23] Jin Z (2021). TRIM59: A potential diagnostic and prognostic biomarker in human tumors. PLoS One.

[24] Han D (2020). The tumor-suppressive human circular RNA CircITCH sponges miR-330-5p to ameliorate doxorubicin-induced cardiotoxicity through upregulating SIRT6, survivin, and SERCA2a. J. Circ. Res..

[25] Han J (2020). A ubiquitin ligase mediates target-directed microRNA decay independently of tailing and trimming. J. Sci..

[26] Vaghi V (2016). Bio-functional surfaces for the immunocapture of AGO2-bound microRNAs. J. Colloids Surf. B Biointerfaces.

[27] Yang C (2022). The ETS1-LINC00278 negative feedback loop plays a role in COL4A1/COL4A2 regulation in laryngeal squamous cell carcinoma. J. Neoplasma.

[28] Wang Y (2018). TRIM59 is a novel marker of poor prognosis and promotes malignant progression of ovarian cancer by Inducing annexin A2 expression. Int. J. Biol. Sci..

[29] Liang M (2020). Cancer-derived exosomal TRIM59 regulates macrophage NLRP3 inflammasome activation to promote lung cancer progression. J. Exp. Clin. Cancer Res..

[30] Li R (2020). TRIM59 predicts poor prognosis and promotes pancreatic cancer progression via the PI3K/AKT/mTOR-glycolysis signaling axis. J. Cell. Biochem..

[31] Zhou WY (2020). Circular RNA: Metabolism, functions and interactions with proteins. J. Mol. Cancer.

[32] Chen Q (2022). Circular RNA ACTN4 promotes intrahepatic cholangiocarcinoma progression by recruiting YBX1 to initiate FZD7 transcription. J. Hepatol..

[33] Li B (2021). circNDUFB2 inhibits non-small cell lung cancer progression via destabilizing IGF2BPs and activating anti-tumor immunity. J. Nat. Commun..

[34] Wang R (2018). EIF4A3-induced circular RNA MMP9 (circMMP9) acts as a sponge of miR-124 and promotes glioblastoma multiforme cell tumorigenesis. J. Mol. Cancer.

[35] Pan G (2019). Upregulation of circMMP9 promotes osteosarcoma progression via targeting miR-1265/CHI3L1 axis. J. Cancer Manag. Res..

[36] Fan HN (2022). METTL14-mediated m(6)A modification of circORC5 suppresses gastric cancer progression by regulating miR-30c-2-3p/AKT1S1 axis. J. Mol. Cancer..

[37] Ando T (2021). Tumor suppressive circular RNA-102450: Development of a novel diagnostic procedure for lymph node metastasis from oral cancer. J. Cancers (Basel).

[38] Lv S (2021). CircRNA GFRA1 promotes hepatocellular carcinoma progression by modulating the miR-498/NAP1L3 axis. J. Sci. Rep..

[39] Zhang Y (2021). circRNA N6-methyladenosine methylation in preeclampsia and the potential role of N6-methyladenosine-modified circPAPPA2 in trophoblast invasion. J. Sci. Rep..

[40] Chen, Z. *et al*. Circular RNA cia-MAF drives self-renewal and metastasis of liver tumor-initiating cells via transcription factor MAFF. *J. Clin. Investig*. **131**, (2021). 10.1172/JCI148020 PMC8483755 34403373

[41] Kim CJ (2018). The transcription factor Ets1 suppresses T follicular helper type 2 cell differentiation to halt the onset of systemic lupus erythematosus. J. Immunity..

[42] Yan M (2022). ETS1 governs pathological tissue-remodeling programs in disease-associated fibroblasts. J. Nat. Immunol..

[43] Dittmer J (2015). The role of the transcription factor Ets1 in carcinoma. J. Semin. Cancer Biol..

[44] Han T (2018). TRIM59 regulates autophagy through modulating both the transcription and the ubiquitination of BECN1. J. Autophagy.

[45] Sun Y (2017). TRIM59 facilitates the proliferation of colorectal cancer and promotes metastasis via the PI3K/AKT pathway. J. Oncol. Rep..

[46] Liu Y (2018). TRIM59 overexpression correlates with poor prognosis and contributes to breast cancer progression through AKT signaling pathway. J. Mol. Carcinog..

[47] Ma S (2019). The interplay between m6A RNA methylation and noncoding RNA in cancer. J. Hematol. Oncol..

[48] Shi B (2022). The role, mechanism, and application of RNA methyltransferase METTL14 in gastrointestinal cancer. J. Mol. Cancer.

[49] Weng H (2022). The mA reader IGF2BP2 regulates glutamine metabolism and represents a therapeutic target in acute myeloid leukemia. J. Cancer Cell..

[50] Shi H (2019). Where, when, and how: Context-dependent functions of RNA methylation writers, readers, and erasers. J. Mol. Cell.

[51] Jiang X (2021). The role of m6A modification in the biological functions and diseases. J. Signal Transduct. Target Ther..

[52] Chen RX (2019). N(6)-methyladenosine modification of circNSUN2 facilitates cytoplasmic export and stabilizes HMGA2 to promote colorectal liver metastasis. J. Nat. Commun..

[53] Liu Z (2021). N(6)-methyladenosine-modified circIGF2BP3 inhibits CD8(+) T-cell responses to facilitate tumor immune evasion by promoting the deubiquitination of PD-L1 in non-small cell lung cancer. J. Mol. Cancer.

[54] Meng W (2019). Aberrant methylation and downregulation of ZNF667-AS1 and ZNF667 promote the malignant progression of laryngeal squamous cell carcinoma. J. Biomed. Sci..

