# Supplementary material for: IGF2BP2-m6A-circMMP9 axis recruits ETS1 to promote TRIM59 transcription in laryngeal squamous cell carcinoma
Source: Sci Rep. 2024 Feb 6;14:3014. doi: 10.1038/s41598-024-53422-4 (PMC10847447; doi:10.1038/s41598-024-53422-4)
Supplement: Supplementary file 1 — Supplementary Information 1. [file 41598_2024_53422_MOESM1_ESM.pdf]

Supplementary Figure The original images of blots

Figure 1A

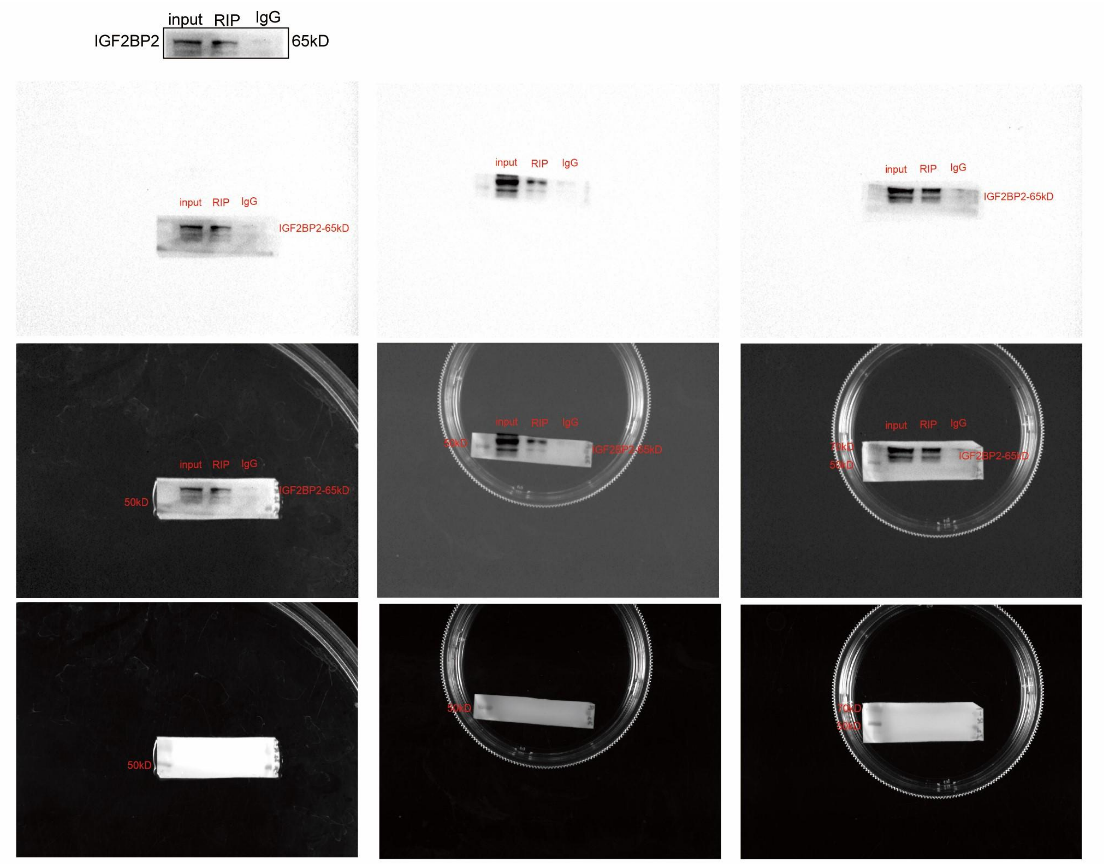

Figure 1B

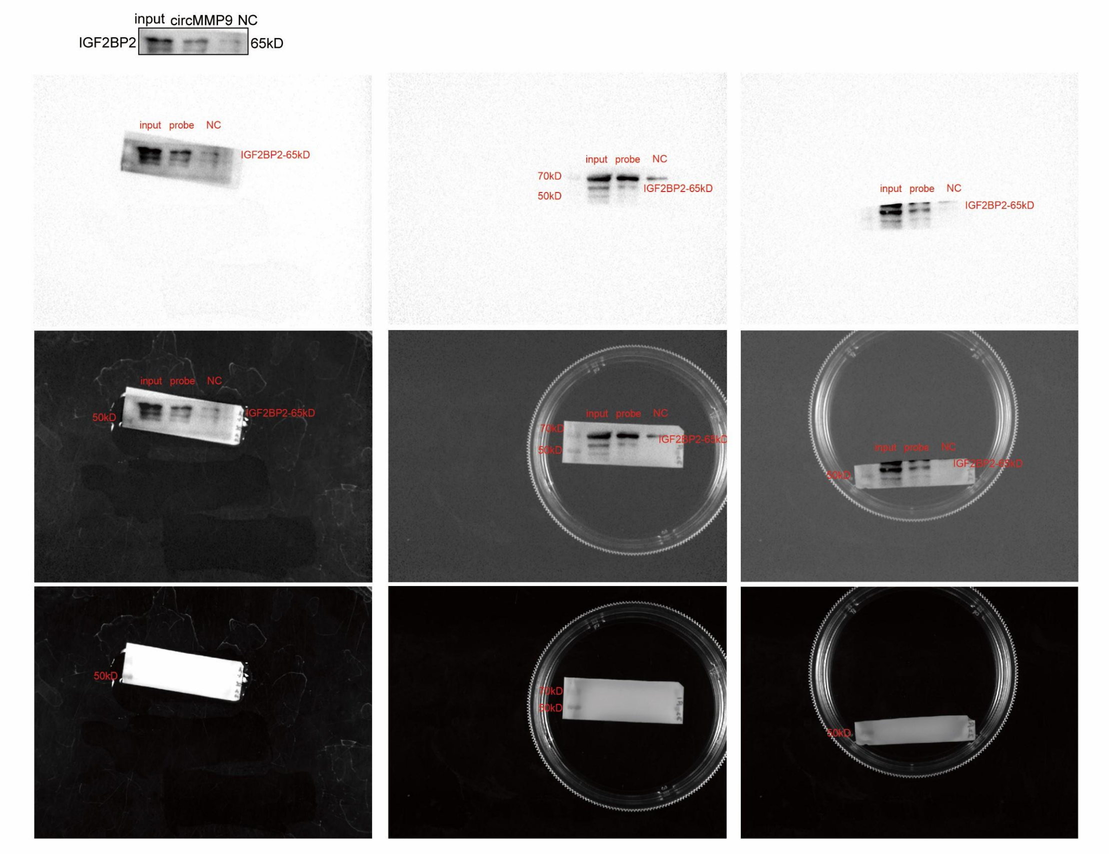

input circMMP9 NC
IGF2BP2
65kD
input probe NC
IGF2BP2-65kD
70kD
50kD
input probe NC
IGF2BP2-65kD
input probe NC
IGF2BP2-65kD
input probe NC
50kD
IGF2BP2-65kD
input probe NC
input probe NC
50kD

Figure 1C

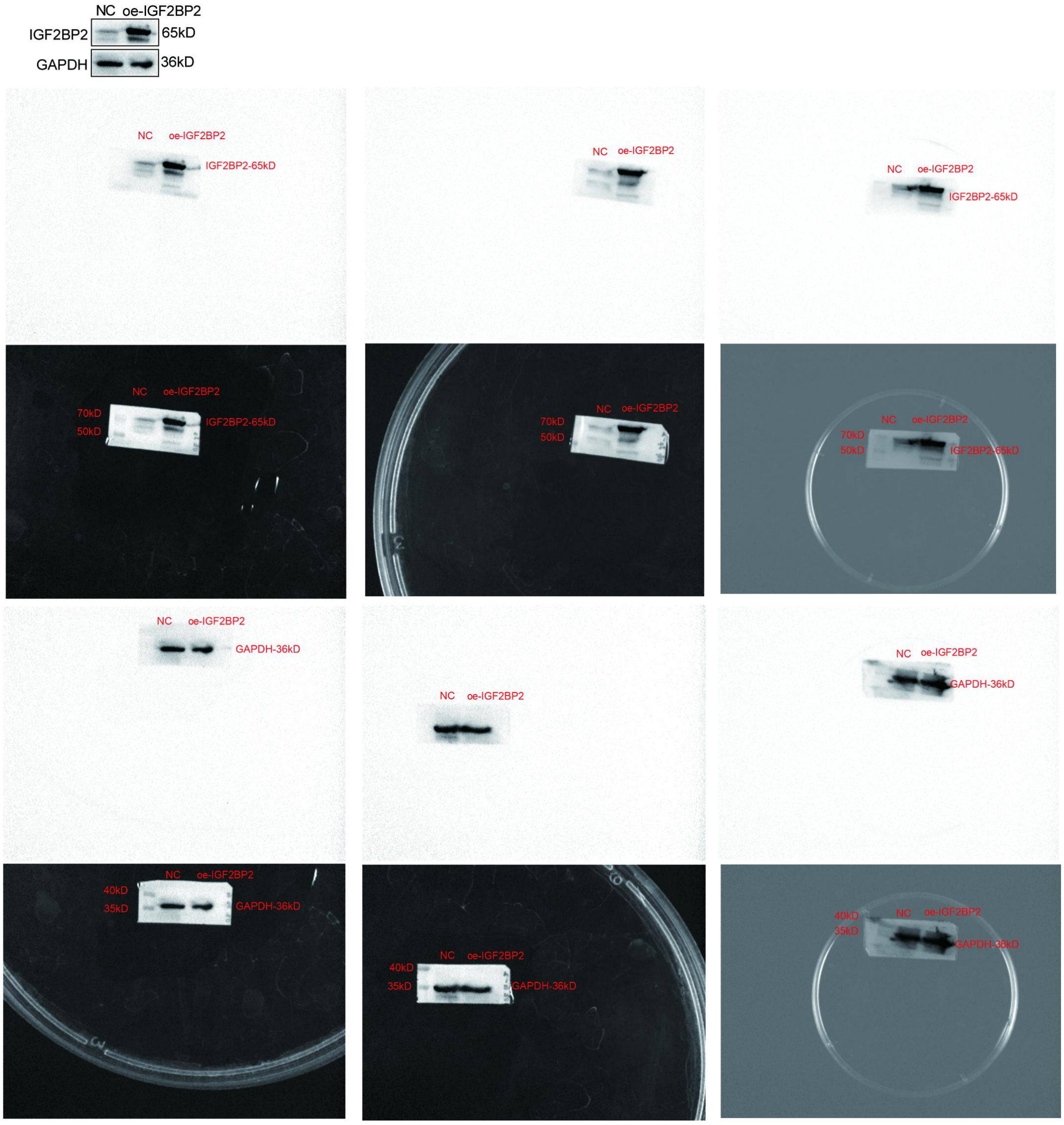
NC
oe-IGF2BP2
IGF2BP2
65kD
GAPDH
36kD
IGF2BP2-65kD
70kD
50kD
GAPDH-36kD
40kD
35kD

Figure 1D

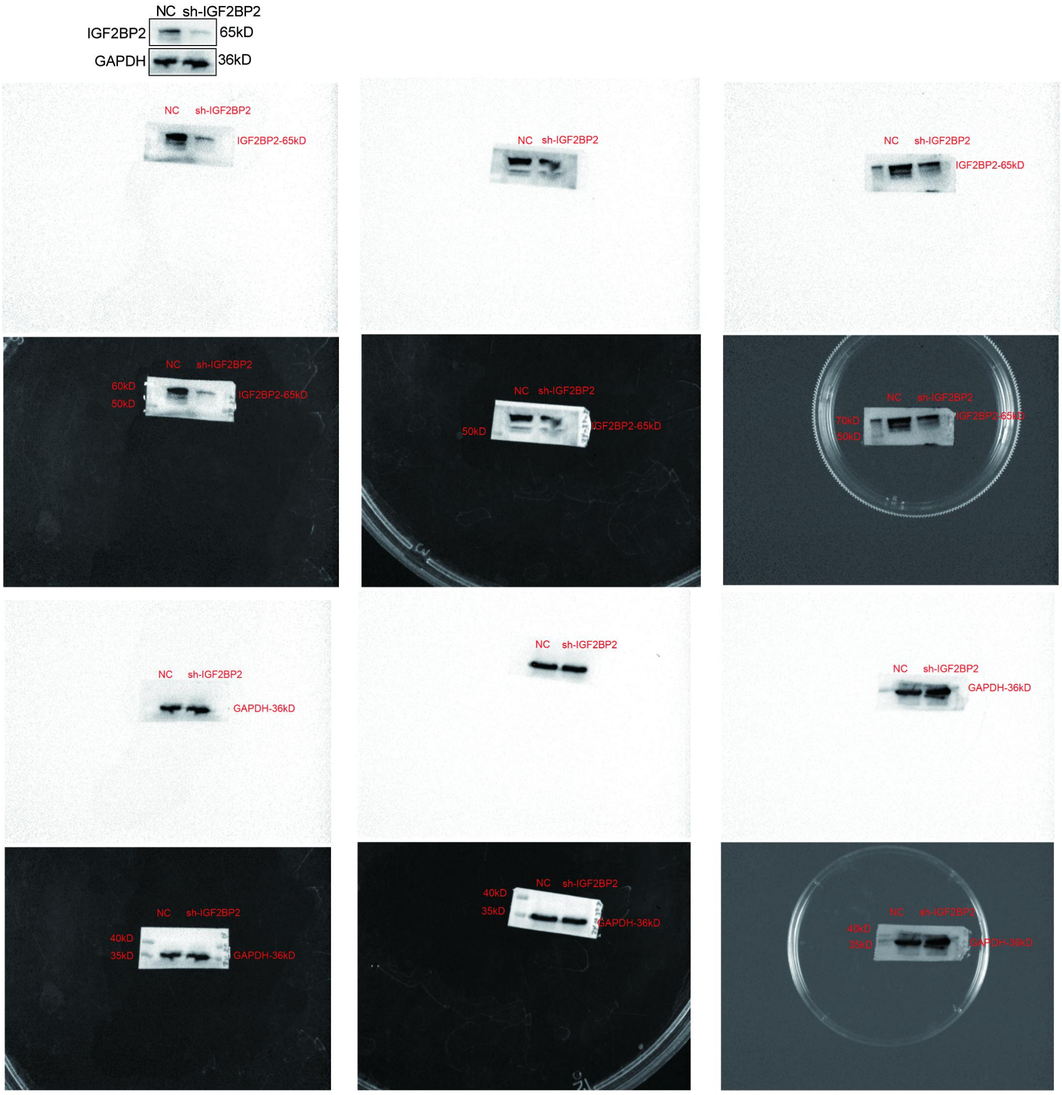
NC
sh-IGF2BP2
IGF2BP2
65kD
GAPDH
36kD
IGF2BP2-65kD
60kD
50kD
70kD
GAPDH-36kD
40kD
35kD

Figure 1E

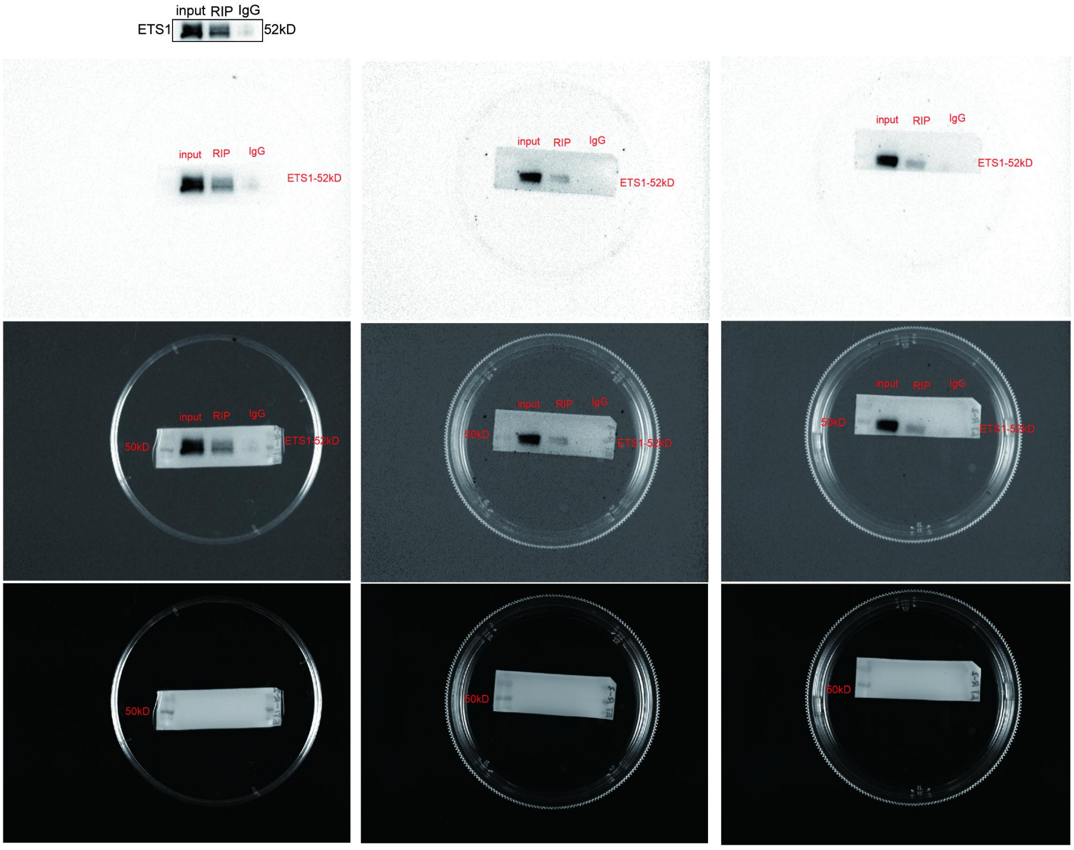

Figure 1F

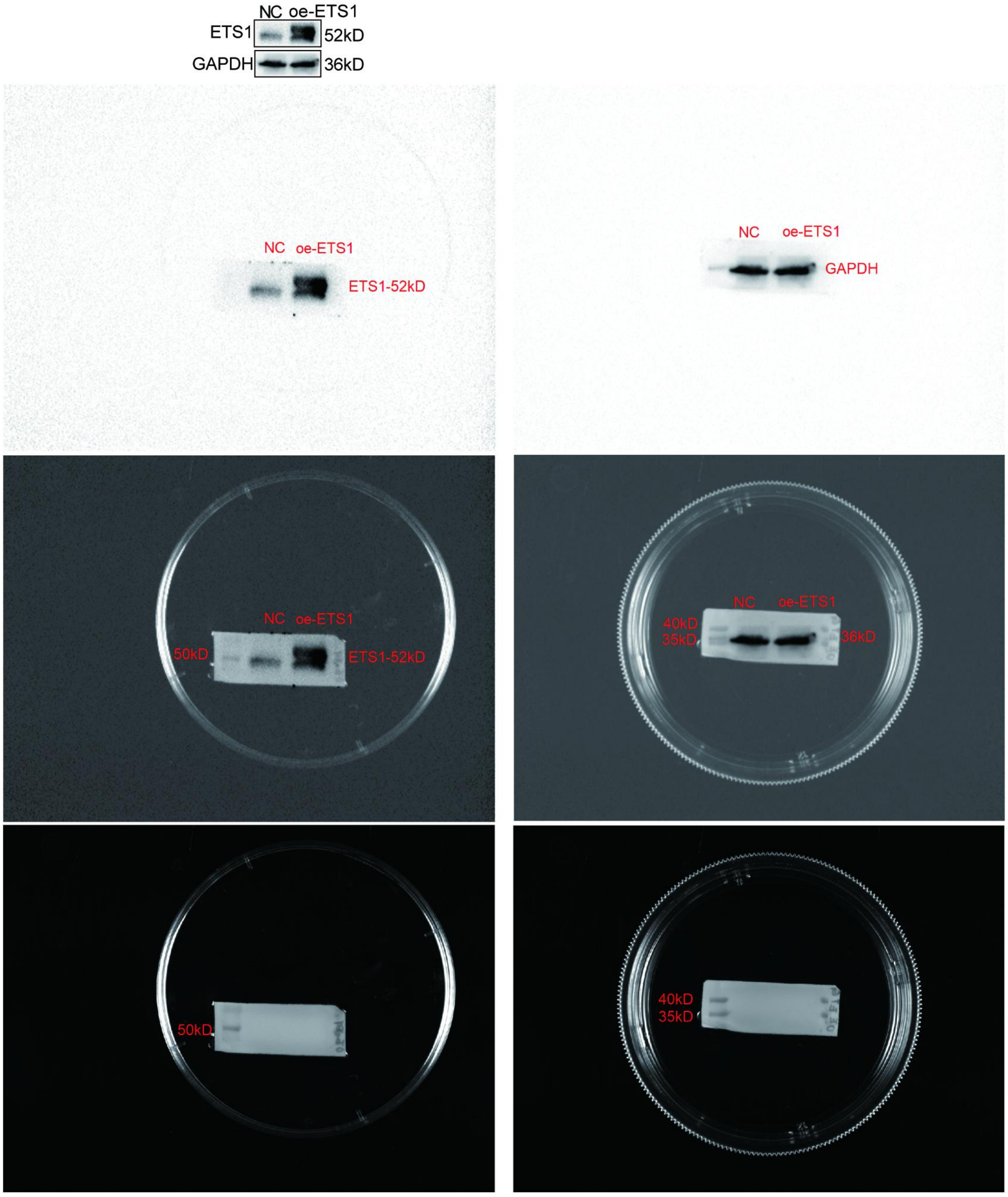

NC
oe-ETS1
ETS1
52kD
GAPDH
36kD
NC
oe-ETS1
ETS1-52kD
NC
oe-ETS1
GAPDH
NC
oe-ETS1
50kD
ETS1-52kD
40kD
35kD
NC
oe-ETS1
36kD
50kD
40kD
35kD

Figure 1G

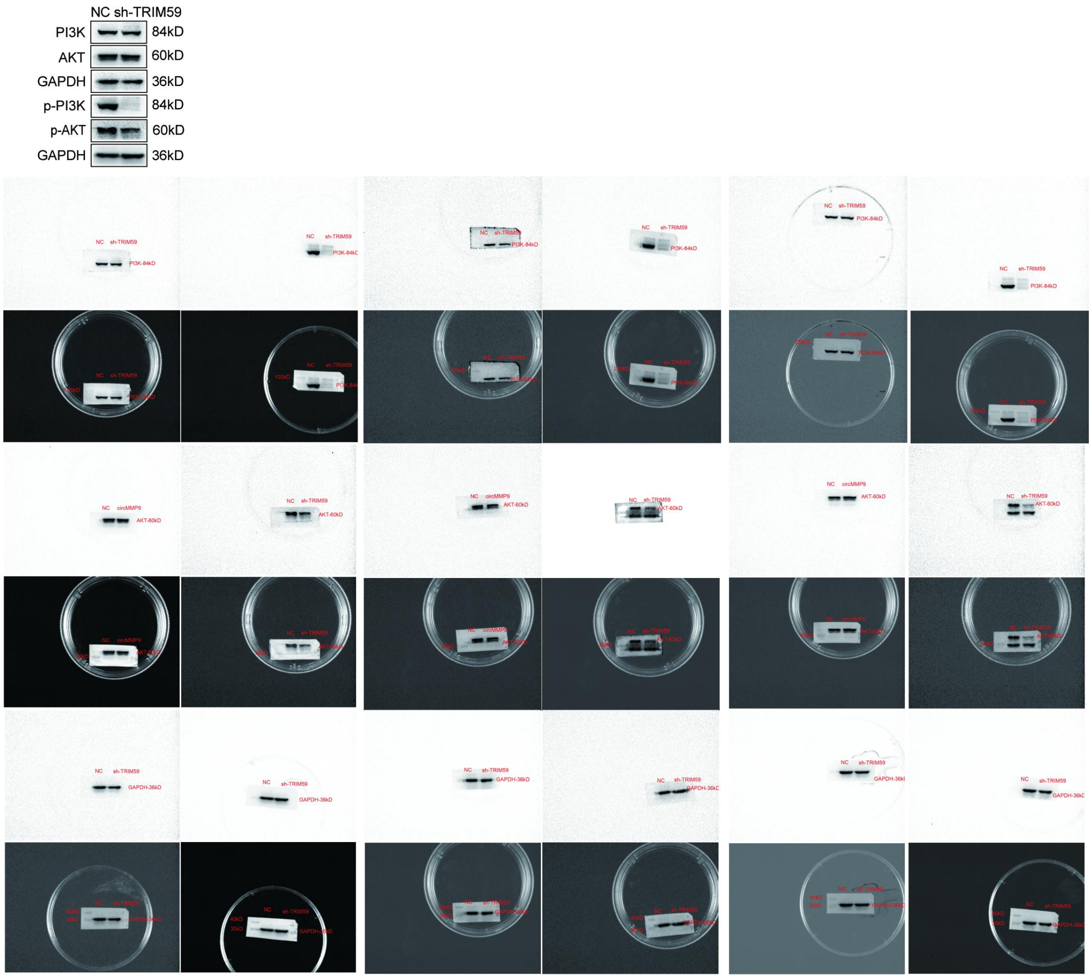
NC sh-TRIM59
PI3K
AKT
GAPDH
p-PI3K
p-AKT
GAPDH
84kD
60kD
36kD
84kD
60kD
36kD

Figure 1H

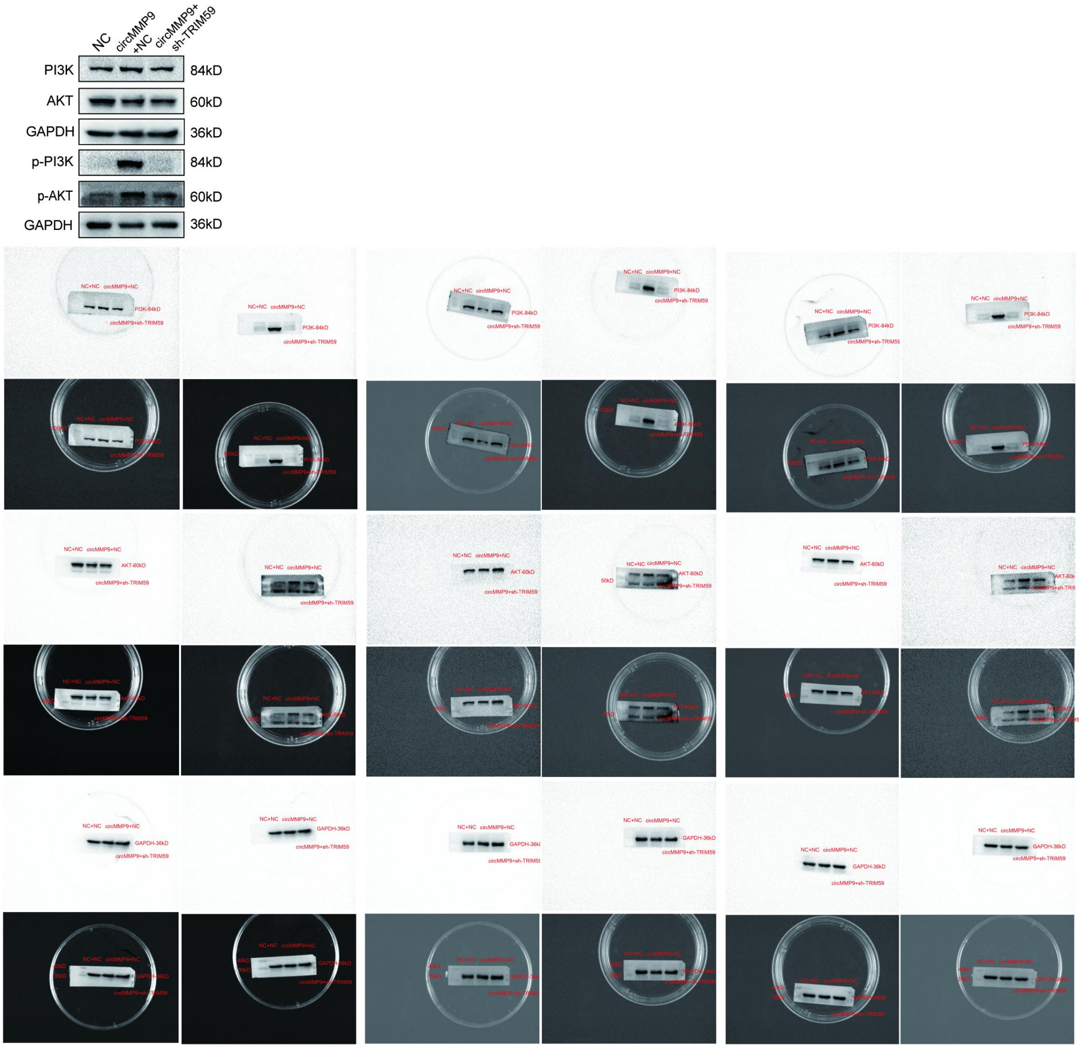
NC
circMMP9 +NC
circMMP9+ sh-TRIM59
PI3K
84kD
AKT
60kD
GAPDH
36kD
p-PI3K
84kD
p-AKT
60kD
GAPDH
36kD
