# Supplementary material for: IGF2BP2-m6A-circMMP9 axis recruits ETS1 to promote TRIM59 transcription in laryngeal squamous cell carcinoma
Source: Sci Rep. 2024 Feb 6;14:3014. doi: 10.1038/s41598-024-53422-4 (PMC10847447; doi:10.1038/s41598-024-53422-4)
Supplement: Supplementary file 6 — Supplementary Table S1. [file 41598_2024_53422_MOESM6_ESM.docx]

**Table S1.** **Clinicopathological characteristics of 50 LSCC samples for qPCR analysis of circMMP9.**

| **Parameters** | **Number of Cases（%）** |
| --- | --- |
| Age(years) |  |
| ＜60 | 17（34） |
| ≥60 | 33（66） |
| Gender |  |
| Female | 0（0） |
| Male | 50（100.0） |
| Smorking |  |
| Yes | 42(84) |
| No | 8(16) |
| Drinking |  |
| Yes | 31(62) |
| No | 19(38) |
| Primary Site |  |
| Glottic | 24（48） |
| Supraglottic | 23（46） |
| Subglottic | 2（4） |
| Transglottic | 1（2） |
| Differentiation |  |
| High | 18（36） |
| Medium | 23（46） |
| Low | 9（18） |
| T Stage**^^[[1]](#footnote-1)^^** |  |
| T1 | 14（28） |
| T2 | 15（30） |
| T3 | 13（26） |
| T4 | 8（16） |
| lymph node metastasis |  |
| N0 | 30（60） |
| N+ | 20（40） |
| Distant metastasis |  |
| M0 | 50（100.0） |
| M1 | 0（0.0） |
| Clinical stage |  |
| I | 8（16） |
| II | 10（20） |
| III | 16（32） |
| IV | 16（32） |

1. TNM Staging is referring to the AJCC 8th edition TNM Staging Criteria. [↑](#footnote-ref-1)
