# Supplementary material for: IGF2BP2-m6A-circMMP9 axis recruits ETS1 to promote TRIM59 transcription in laryngeal squamous cell carcinoma
Source: Sci Rep. 2024 Feb 6;14:3014. doi: 10.1038/s41598-024-53422-4 (PMC10847447; doi:10.1038/s41598-024-53422-4)
Supplement: Supplementary file 7 — Supplementary Table S2. [file 41598_2024_53422_MOESM7_ESM.docx]

**Supplementary S2. Sequences of primer, shRNA and RNA pull down probe used in this study**

| Sh-IGF2BP2-F | GATCCAGTGAAGCTGGAAGCGCATATTTCAAGACGTTTAATGGTTGCCTGTAATGTTTTTTTGTCGACA |
| --- | --- |
| Sh-IGF2BP2-R | AGCTTGTCGACAAAAAAAGTGAAGCTGGAAGCGCATATCGTCTTGAATTTAATGGTTGCCTGTAATGTG |
| Sh-TRIM59-F | GATCCACATTACAGGCAACCATTAAATTCAAGACGTTTAATGGTTGCCTGTAATGTTTTTTTGTCGACA |
| Sh-TRIM59-R | AGCTTGTCGACAAAAAAACATTACAGGCAACCATTAAACGTCTTGAATTTAATGGTTGCCTGTAATGTG |
| Sh-circMMP9-F | GATCCTTTAGTCCTCGCCCTGAACCCTTCCTGTCAGAGGTTCAGGGCGAGGACTAAATTTTTG |
| Sh-circMMP9-R | AATTCAAAAATTTAGTCCTCGCCCTGAACCTCTGACAGGAAGGGTTCAGGGCGAGGACTAAAG |
| CircMMP9-F | TGTAAATCCCCACTGGGACC |
| CircMMP9-R | TTCAGGGCGAGGACTAAAGG |
| CircMMP9-ASO | TTTAGTCCTCGCCCTGAACC |
| MMP9-F | GGCAGGGACAGTTGCTTCT |
| MMP9-R | TGTACCGCTATGGTTACACTCG |
| ETS1-F | GATAGTTGTGATCGCCTCACC |
| ETS1-R | GTCCTCTGAGTCGAAGCTGTC |
| IGF2BP2-F | GTAAAGTGGAATTGCATGGGA |
| IGF2BP2-R | CAAAAGTCCATCCAACACCTC |
| ChIP P1-F | TTGACAGGAAGTTACAAAGA |
| ChIP P1-R | CTGTTTCATGGTGGTAAATA |
| ChIP P2-F | CTGCCCCAGCCTCCCAACTAG |
| ChIP P2-R | CCAACATGGTGAAACCCCGTC |
| ChIP P3-F | TGGGTGTTCCCTGAGAATGTG |
| ChIP P3-R | GGGCCTCCTGCTATGCTGTG |
| ChIP P4-F | TGAATGTTGCCTGCAGACTGC |
| ChIP P4-R | GCCCTCCTCTGCCCCGAAG |
| P1-F | ACGCGTGCTAGCCCGGGCCTTGGCCTCCAGAAAGTG |
| P1-R | GTACCGGAATGCCAAGCTTCCAGCGTAAGGCTTGAG |
| P2-F | ACGCGTGCTAGCCCGGGCACGATGGAAGACACTGCG |
| P2-R | GTACCGGAATGCCAAGCTGAGAGCCGCCCCGAGT |
| P3-F | ACGCGTGCTAGCCCGGGCGACAGGAGAGGGAAGCGGTG |
| P3-R | GTACCGGAATGCCAAGCTCGGGGCGGGGCCTCCTGCTA |
| P4-F | ACGCGTGCTAGCCCGGGCTTGACAGGAAGTTACAAAGATG |
| P4-R | ACGCGTGCTAGCCCGGGCTTGACAGGAAGTTACAAAGATG |
| TRIM59-F | AAGATCCTCGTGTACTGCCAT |
| TRIM59-R | CAATGCCAGTTGGAGCAATTTC |
| GAPDH-F | AGGTGAAGGTCGGAGTCAACG |
| GAPDH-R | AGGGGTCATTGATGGCAACA |
| U6-F | GCTTCGGCAGCACATATACTAAAAT |
| U6-R | CGCTTCACGAATTTGCGTGTCAT |
| MeRIP-qPCR-F | TGTAAATCCCCACTGGGACC |
| MeRIP-qPCR-R | TTCAGGGCGAGGACTAAAGG |
| CircMMP9-probe | GAGGACTAAAGGTTAGAGAA-/3bio/ |
| NC-probe | AATGCAAGCTGGTAATCTGA-/3bio/ |
