# Supplementary material for: IGF2BP2-m6A-circMMP9 axis recruits ETS1 to promote TRIM59 transcription in laryngeal squamous cell carcinoma
Source: Sci Rep. 2024 Feb 6;14:3014. doi: 10.1038/s41598-024-53422-4 (PMC10847447; doi:10.1038/s41598-024-53422-4)
Supplement: Supplementary file 8 — Supplementary Table S3. [file 41598_2024_53422_MOESM8_ESM.docx]

**Supplementary Table S3 Antibodies used in this study**

| **Antibodies** | **Source** | **Identifier** |
| --- | --- | --- |
| Anti-m6A | Abcam | Cat#ab208577 |
| Anti-GAPDH | Proteintech | Cat# 10494-1-AP |
| Anti-IGF2BP2 | Proteintech | Cat#11601-1-AP |
| Anti-ETS1 | Cell Signaling | # 14069 |
| Anti-PI3K | HUABIO | EM1701-62 |
| Anti-AKT | Cell Signaling | #4691 |
| Anti-p-PI3K | Gene Tex | GTX132597 |
| Anti-p-AKT | Cell Signaling | #4060 |
| Anti-rabbit IgG (H+L) | Proteintech | Cat#30000-0-AP |
